# Genome-wide identification and in silico characterization of major RNAi gene families in date palm (*Phoenix dactylifera*)

**DOI:** 10.1186/s12863-024-01217-x

**Published:** 2024-03-15

**Authors:** Darun Naim, Asif Ahsan, Ahmed Imtiaj, Nurul Haque Mollah

**Affiliations:** 1 Bioinformatics Lab, Department of Statistics, Faculty of Science, University of Rajshahi, 6205 Rajshahi, Bangladesh; 2 Department of Botany, Faculty of Biological Sciences, University of Rajshahi, 6205 Rajshahi, Bangladesh

**Keywords:** Date palm, RNAi genes, Characterization, Functional analysis, Integrated bioinformatics analysis

## Abstract

**Background:**

Dates contain various minerals that are essential for good health. The major RNA interference (RNAi) gene families play a vital role in plant growth and development by controlling the expression of protein-coding genes against different biotic and abiotic stresses. However, these gene families for date palm are not yet studied. Therefore, this study has explored major RNAi genes and their characteristics in date palm.

**Results:**

We have identified 4 PdDCLs, 7 PdAGOs, and 3 PdRDRs as RNAi proteins from the date palm genome by using AtRNAi genes as query sequences in BLASTp search. Domain analysis of predicted RNAi genes has revealed the Helicase_C, Dicer_dimer, PAZ, RNase III, and Piwi domains that are associated with the gene silencing mechanisms. Most PdRNAi proteins have been found in the nucleus and cytosol associated with the gene silencing actions. The gene ontology (GO) enrichment analysis has revealed some important GO terms including RNA interference, dsRNA fragmentation, and ribonuclease_III activity that are related to the protein-coding gene silencing mechanisms. Gene regulatory network (GRN) analysis has identified PAZ and SNF2 as the transcriptional regulators of PdRNAi genes. Top-ranked 10 microRNAs including Pda-miR156b, Pda-miR396a, Pda-miR166a, Pda-miR167d, and Pda-miR529a have been identified as the key post-transcriptional regulators of PdRNAi genes that are associated with different biotic/abiotic stresses. The *cis*-acting regulatory element analysis of PdRNAi genes has detected some vital *cis*-acting elements including ABRE, MBS, MYB, MYC, Box-4, G-box, I-box, and STRE that are linked with different abiotic stresses.

**Conclusion:**

The results of this study might be valuable resources for the improvement of different characteristics in date palm by further studies in wet-lab.

**Supplementary Information:**

The online version contains supplementary material available at 10.1186/s12863-024-01217-x.

## Introduction

RNA interference (RNAi) is a common mechanism in which numerous forms of RNA molecules affect the expression of protein-coding genes at the transcriptional or post-transcriptional levels that influences different characteristics of plant including development, growth, stress responses, and antimicrobial defense [1]. This mechanism occurs in three pathways known as micro-RNA (miRNA), endogenous small interfering RNA (siRNA), and PIWI-interacting RNA (piRNA) biogenesis [2]. It is used as a tool for controlling gene expression and investigating gene function on a whole-genome scale [3–8]. The major RNAi genes are known as argonaute (AGO), RNA-dependent RNA polymerase (RDR), and Dicer-like (DCL) genes/proteins [9]. The RNAi mechanism is initiated by partially double-stranded stem-loop RNA or double-stranded RNA (dsRNA), which is cleaved into 21-24-nt short RNA (sRNA) duplexes by DCL [10]. Dicer endonucleases is a central component in the biogenesis of sRNA molecules [11]. AGO is an essential part of the RNA-induced silencing complex (RISC) [12] and RDR plays a key role in synthesizing dsRNAs from an RNA template [13]. The number of RNAi genes in the *DCL*, *AGO*, and *RDR* families in plants varies by species variations, such as 20 genes in *Arabidopsis* [14] and cucumber [15], 51 genes in Brassica species [16], 28 genes in maize [17] and tomato [18], 32 genes in rice [19], 19 genes in barley [20], 38 genes in foxtail millet [21], 22 genes in grapevine [22] and pepper [23], 25 genes in sweet orange [9], 36 genes in sugarcane [24], and 31 genes in tea [25] have been identified [26].

RNAi genes perform different biological activities in plants by controlling microbial (RNA and DNA viruses, viroids, insects, and, more recently, fungal diseases) stresses, expression of protein coding genes and chromatin condensation into heterochromatin [27–36]. Most plants contain four DCLs, with *DCL1* causing microRNA synthesis and *DCL2, DCL3*, and *DCL4* essential for 22-, 24-, and 21-nucleotide small interfering RNA (siRNA) biogenesis, respectively [37]. The *DCL2* and *DCL4* have roles in the creation of transacting siRNA, while *DCL2* is primarily responsible for the generation of various-sized secondary siRNAs [38]. The integrated actions of *DCL2*, *DCL3*, and *DCL4* play a vital role in disease response and protection [39]. The AGO1 is sensitive to virus attacks, confirming the concept that in plants, PTGS represents a defense system against viruses [40]. The *AGO2* gene is activated in response to the presence of turnip crinkle virus (TCV) and cucumber mosaic virus (CMV) [41]. The *RDR6* plays a significant role in RNA silencing processes [42] and all RDRs have an impact on plant development and stress responses [43]. The exogenously injected *VICE12*-dsRNA targets the *VICE12* gene of apple scab fungus to inhibit its growth and conidial spore formation via the RNAi mechanism [44]. The transgenic tomato plant has been developed by applying the RNAi mechanism using exogenously injected *CopE/TLR6*-dsRNAs, resulting in a greater mortality rate for western flower thrips (WFT) than wild-type tomato plants when fed to WFT [45].

The date palm trees are ancient Asian and African trees that are farmed for their delicious, edible and medicinal value [46]. One of the most nutritious fruits in the Middle East and North Africa is the date fruit [47]. There are more than 100 million date palm trees in the world [48]. It is well-known for its significant nutrients, dietary fiber, natural antioxidants and sources of rich bioactive compounds that are useful in the treatment of neurological diseases and cancer [49]. Moreover, date fruits are source of carbohydrates, alkaloids, fatty acids (palmitic, linoleic, lauric, and stearic acid), vitamins, carotenoids, flavonoids, polyphenolic compounds, and tannins, as well as other nutrients such as calcium, magnesium, potassium, and phosphorus [50]. Date palm harvest in the world has risen from 4.60 to 8.52 million tons from 1994 to 2018 (FAO. 2020). Date palm pollen is high in bioactive substances with phytochemical and nutritional properties, which could boost anti-infertility capabilities [51]. Date fruit flesh, peel, and pits have anti-mutagenic, hepatoprotective, anti-inflammatory, anti-diabetic, anti-bacterial, anti-viral, anti-fungal, anti-tumor, nephroprotective, heart disease protective, and anti-cancer characteristics [26]. Worldwide, up to 50% newly planted date palm trees and fruits are lossing due to the pathogenic infections and other environmental stresses [52–54]. To improve this situation, RNAi genes may play a vital role according to the literature review. However, so far, there has been no information regarding these major RNAi gene families in the economically important date palm. Therefore, this study aimed to gather extensive information on the major RNAi gene families (*DCL*, *AGO*, and *RDR* ) that may contribute to the growth and development of date palm.

## Materials and methods

### The data source and descriptions

To explore PdRNAi proteins (DCL, AGO, and RDR) from the date palm (*Phoenix dactylifera*) genome, we have considered its genome/proteome sequences from the National Center for Biotechnology Information (NCBI) database [55, 56] with GenBank accession number: GCA_009389715.1 (NCBI taxonomy ID: 42,345, BioSample: SAMN05011615, weblink: https://www.ncbi.nlm.nih.gov/genome/?term=Phoenix+dactylifera). This dataset has been utilized in comparative genomics study to identify male (Y) and female (X) chromosomes in date palm [57]. It has also been utilized in transcriptomics and genome-wide association studies [58, 59]. In this study, this genome dataset has been utilized to explore PdRNAi proteins by BLASTP search with the query sequences of *A. thaliana* RNAi (AtRNAi) proteins. Total 17 of 20 AtRNAi proteins (4 AtDCL, 10 AtAGO, and 3 AtRDR) sequence has been downloaded from the *Arabidopsis* Information Resource (TAIR) database that has been developed by P. Lamesch et al. (https://www.arabidopsis.org/) [60].

### Integrated bioinformatics analyses

The integrated bioinformatics studies include BLASTP search, multiple sequence alignment (MSA), phylogenetic tree modeling, functional domain analysis, exon-intron structure of predicted PdRNAi of date palm, subcellular location, GO analysis, TFs analysis, CAREs analysis and miRNA analysis.

### Identification of PdRNAi proteins

#### Exploring PdRNAi proteins by BLASTP serach

The date palm genome from the NCBI database has been analyzed using the basic local alignment search algorithm for proteins (BLASTP) [61] to explore PdRNAi genes. The protein sequences of RNAi genes have been retrieved using query coverage (≥ 50%) and E-values (0.00) [9, 62]. However, only the top-scorer aligned sequences have been considered as the sequences of PdRNAi genes. The genomic length, protein ID, CDS length, and encoded protein length of PdRNAi genes have been retrieved from the NCBI database. The molecular weight, pI, and GRAVY of PdRNAi protein sequences have been calculated using ExPASy [63].

### Phylogenetic tree construction among PdRNAi and AtRNAi proteins to fix the names of PdRNAi proteins

The MSAs of the expected PdRNAi protein sequences have been constructed using the Clustal-W [64] method and the MEGAX [65] package. By performing phylogenetic tree analysis on the PdRNAi genes using the Neighbor-joining technique [66], 1,000 bootstrap repetitions [67] have been utilized to confirm the evolutionary connection. The evolutionary distances have been determined using the equal input technique [68].

### Charaterization of PdRNAi

#### Conserved domains and motifs analysis of PdRNAi proteins with respect to AtRNAi proteins

Pfam has been used to search conserved domains in protein sequences [69]. The TBtools (a Toolkit for Biologists integrating various biological data-handling tools) has been used to display the Pfam’s result [70]. The conserved domains have been retrieved from the Pfam database to examine the PdRNAi proteins functional domains. We have investigated the conserved motifs in all of the anticipated PdDCL, PdAGO, and PdRDR proteins using the Multiple Expectation Maximization for Motif Elicitation (MEME) webtool [71]. The TBtools have been used to display the motif’s result [70].

### PdRNAi genes structures with respect to AtRNAi genes

We have been used the Gene Structure Display Server (GSDS 2.0) to determine the gene structure of the anticipated PdRNAi genes [72]. The structure of the targeted genes of date palm has been compared with the *A. thaliana* gene structure through the exon-intron composition.

### Sub-cellular localizations of PdRNAi proteins with respect to AtRNAi proteins

The subcellular location of reported PdRNAi proteins in the cell has been investigated by subcellular location analysis. The web tool WoLF PSORT has been used to predict the subcellular location of the targeted proteins [73]. We have been used the TBtools program to display the result [70].

### Functional enrichment analysis of PdRNAi proteins

The Gene Ontology (GO) analysis has been carried out using an online database PlantTFDB to confirm the participation of PdRNAi proteins in biological processes (BPs) and molecular functions (MFs) terms [74]. The *p*-values has been calculated using Fisher’s exact test with Benjamini-Hochberg adjustments. We have considered a GO term with a *p*-value < 0.05 as statistically significant.

### PdRNAi gene regulatory network analysis

**Fig. 1:**
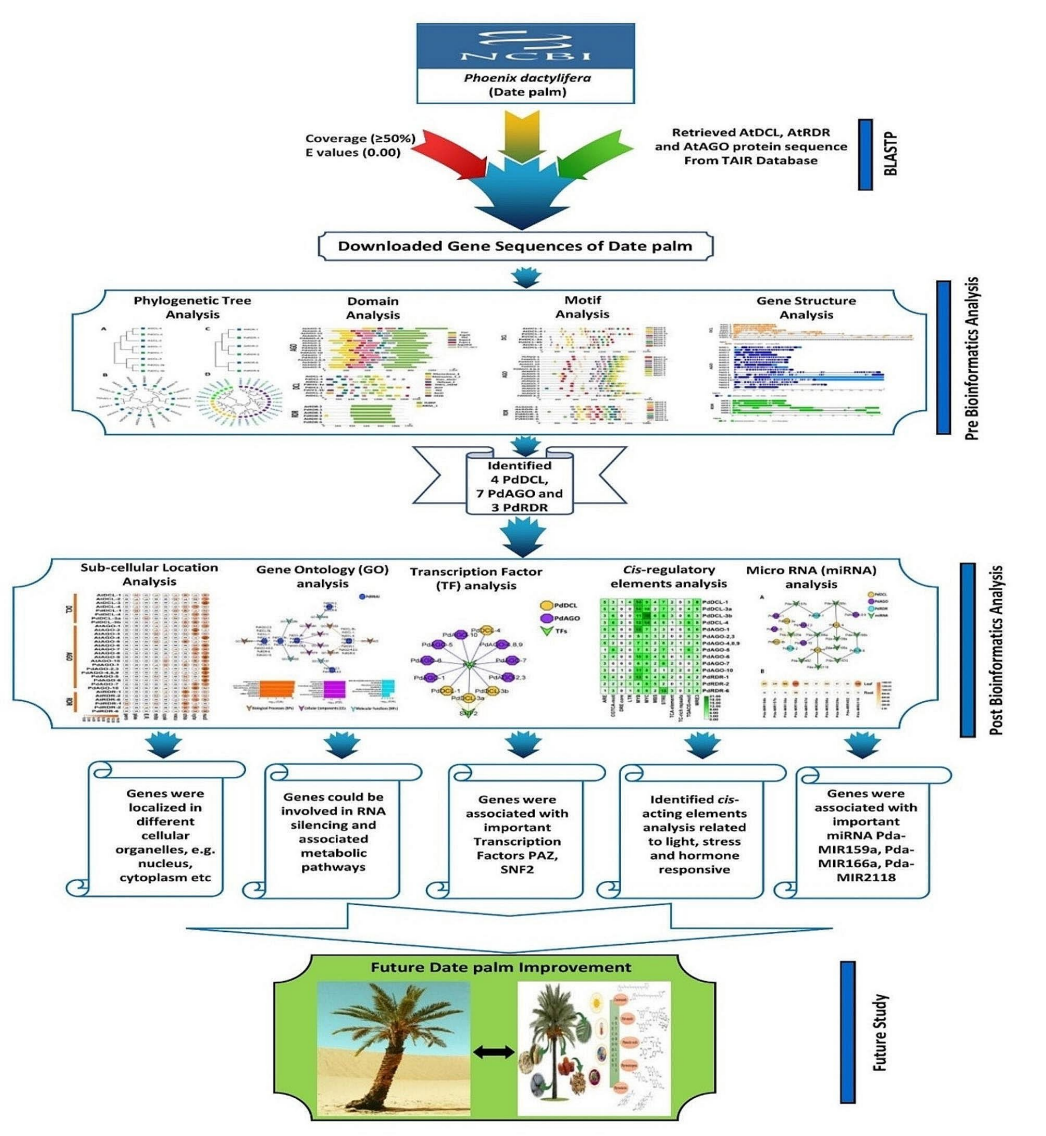
The outline of this study

In this study, the associated TF families of the PdRNAi genes in date palm has been analyzed using PlantTFcat, a widely used plant transcription factor database [75]. The regulatory network and sub-network has been constructed by integrating TFs and PdRNAi genes and analyzed using Cytoscape 3.9.0 [76]. From the network, we have identified the hub genes and related major hub TFs of PdRNAi based on the degree of connectivity. *Cis*-element analysis has been done by Plant CAREs [77] database. We have used the Plant miRNA ENcyclopedia (PmiREN) to download date palm mature_miRNA_expression and mature_miRNA_sequence [78]. After that, we have used Plant sRNA target (psRNATarget) [79] to identify the mature_miRNA_expression and mature_miRNA_sequence IDs that corresponded to our predicted PdRNAi genes. The TBtools program has been used to display this data [70]. The detailed pipeline of this study is given in Fig. 1.

## Results

### Identification of PdRNAi proteins

We have identified four DCL, seven AGO, and three RDR proteins from the date palm genome by top-scorer aligned sequences (Table 1). Therefore, we have considered these 14 PdRNAi proteins for further investigation. For the convenience of presentation, we have denoted *P. dactylifera* RNAi protein families as PdDCL, PdAGO, and PdRDR and *A. thaliana* RNAi protein families as AtDCLs, AtAGOs, and AtRDRs.

**Table 1:** Basic information of predicted DCL, AGO, and RDR protein families of P. dactylifera (Pd)

Serial	Protein name	Protein ID	Chromosomal location	CDS (bp)	No of Ex./Int.	Protein			
						Len (aa)	M.W. (D)	pI	GRAVY
**DCL**									
1	PdDCL-1	XP_008783544.2	chr5; NC_052396.1 (7541438.7558386)	6275	19:18	1932	217043.2	6	-0.43
2	PdDCL-3a	XP_038975056.1	chrUn; NW_024067910.1 (148343.226673)	7331	28:29	1824	206421.7	6.3	-0.3
3	PdDCL-3b	XP_038975924.1	chrUn; NW_024068062.1 (66741.145337)	2486	26:25	714	184023.2	7.8	-0.157
4	PdDCL-4	XP_008793257.2	chr9; NW_008246593.1 (751820.772764	5281	25:24	1510	169709.8	6.3	-0.138
**AGO**									
1	PdAGO-1	XP_026666285.1	chrUn; NW_024067681.1 (656667.671825)	3725	23:22	1109	117304.5	9.4	-0.44
2	PdAGO-2,3	XP_026665120.2	chr16; NC_052407.1 (10638701.10642469)	3242	3:2	970	108819.8	9.2	-0.462
3	PdAGO-4,8,9	XP_008805343.1	chr1; NC_052392.1 (27566908.27575426)	2980	25:24	852	102903.6	8.9	-0.407
4	PdAGO-5	XP_038990444.1	chr16; NC_052407.1 (380550.387158)	3425	22:21	991	110499.3	9.3	-0.523
5	PdAGO-6	XP_026664236.2	chr1; NC_052392.1 (23234962.23244557)	3283	24:23	906	101653.8	9.3	-0.316
6	PdAGO-7	XP_008787212.2	chr6; NC_052397.1 (14183313.14187911)	3894	4:3	1013	114837.4	9.3	-0.419
7	PdAGO-10	XP_038987839.1	chr11; NC_052402.1 (23479152.23494653)	4236	26:25	973	109504.7	9.3	-0.426
**RDR**									
1	PdRDR-1	XP_008812977.2	chrUn; NW_024067889.1 (372284.383973)	3954	6:5	1131	129394.4	8.3	-0.309
2	PdRDR-2	XP_008780950.1	chr1; NC_052392.1 (20350773.20361462)	3670	4:3	1117	126903.6	7	-0.255
3	PdRDR-6	XP_008778879.2	chr2; NC_052393.1 (23599135.23610932)	4231	3:2	1198	135654.2	6.3	-0.287

N.B:The protein names, protein ID, chromosomal location, CDS length, protein length (aa) have been collected from NCBI database. The molecular weight, isoelectric point (pI), and grand average of hydropathicity (GRAVY) values have been collected from the ExPASy. Molecular weights (M. W.) have been measured in Daltons (D) and “aa” means amino acid

**Fig. 2:**
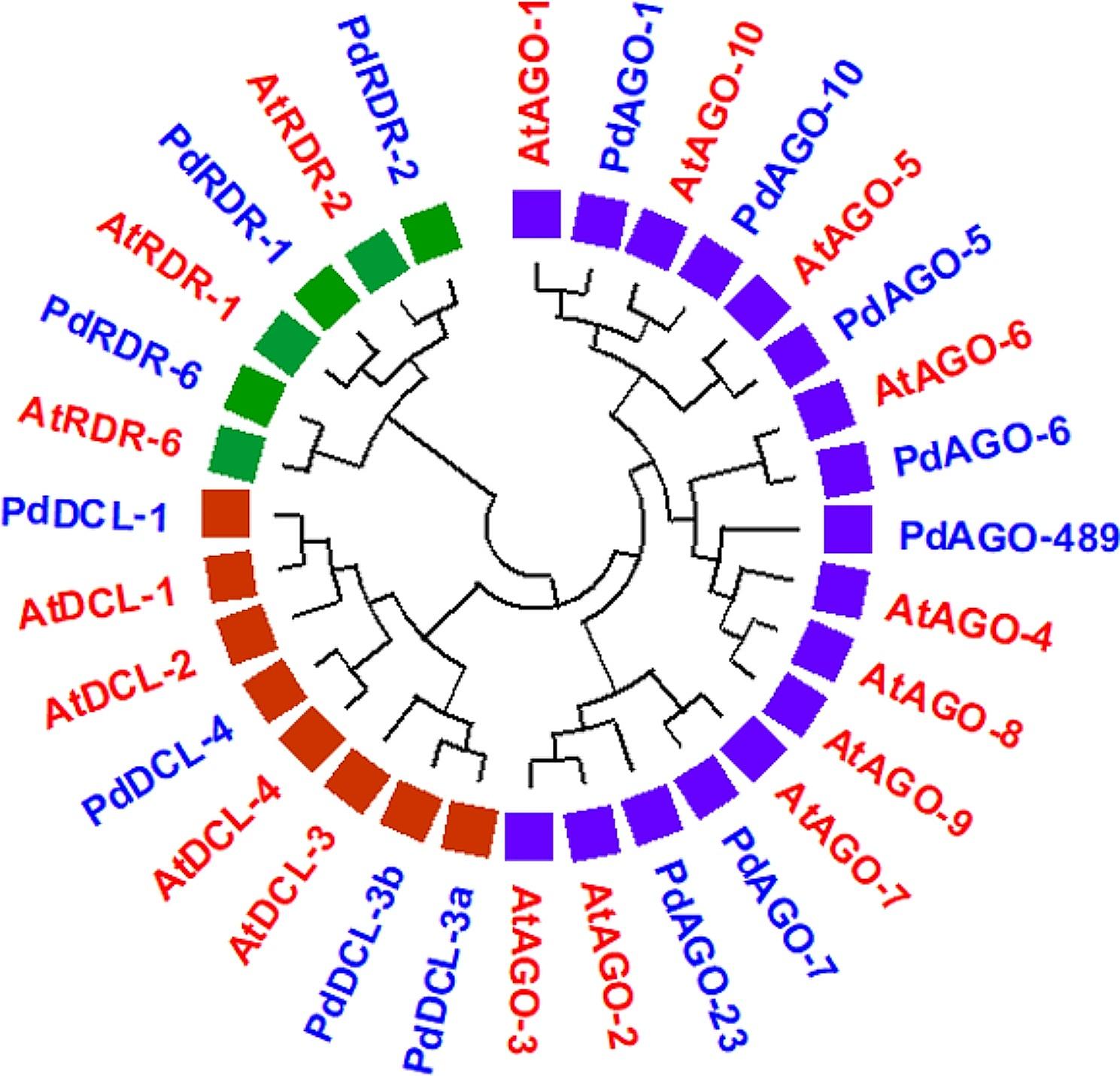
The combined phylogenetic tree. In this tree, the PdRNAi (PdDCLs, PdAGOs, and PdRDRs) and the model plant AtRNAi (AtDCLs, AtAGOs, AtRDRs) proteins have been represented by blue and red, respectively. Here DCLs, AGOs, and RDRs families have been represented by orange, purple, and green circles, respectively

Thereafter, we performed MSAs of PdRNAi and AtRNAi proteins to construct the phylogenetic tree. After that, we have built the phylogenetic tree based on the aligned sequences of the PdRNAi and AtRNAi protein families (Fig. 2). The names of 14 PdRNAi proteins have been denoted as 4 PdDCLs proteins (PdDCL-1, PdDCL-3a, PdDCL-3b, PdDCL-4), 7 PdAGOs proteins (PdAGO-1, PdAGO-2,3, PdAGO-4,8,9, PdAGO-5, PdAGO-6, PdAGO-7, PdAGO-10), and 3 PdRDRs proteins (PdRDR-1, PdRDR-2, PdRDR-6) based on the sequence similarity to AtRNAi proteins.

The lengths of PdDCL genes are ranging from CDS 2486 (*PdDCL-3b*) to 7331 (*PdDCL-3a*) bp and their protein lengths are ranging from 714 to 1824 amino acids (Table 1). The lengths of PdAGO genes are ranging from CDS 2980 (*PdAGO-4,8,9*) to 4236 (*PdAGO-10*) bp and their protein lengths are ranging from 852 to 973 amino acids (Table 1). The lengths of PdRDR genes are ranging from CDS 4231 (*PdRDR-6*) to 3670 (*PdRDR-2*) bp and their protein lengths are ranging from 1117 to 1198 amino acids (Table 1). The isoelectric point (pI) values of the PdDCL genes revealed that they are slightly acidic, with only the *PdDCL-3b*, which is slightly alkaline, with a maximum pI value of 7.8. The PdAGO genes pI values showed that they are alkaline. The pI values of the PdRDR genes demonstrated that they are nearly neutral.

The PdDCL proteins have a grand average hydropathicity index (GRAVY) or peptides hydrophobicity value that are ranging from − 0.138 (PdDCL-4) to -0.43 (PdDCL-1). The GRAVY value of the PdAGO proteins are ranging from − 0.316 (PdAGO-6) to -0.523 (PdAGO-5), whereas for PdRDR proteins are from − 0.255 (PdRDR-2) to -0.309 (PdRDR-1). We have also investigated the phylogenetic relationship between PdRNAi and *Oryza sativa* RNAi (OsRNAi) proteins. We have observed that PdDCLs, PdAGOs, and PdRDRs belong to the same clusters of OsDCLs, OsAGOs, and OsRDRs, respectively (Fig. S1).

### Charaterization of PdRNAi proteins

#### Conserved domains and motifs of PdRNAi proteins with respect to AtRNAi proteins

**Fig. 3:**
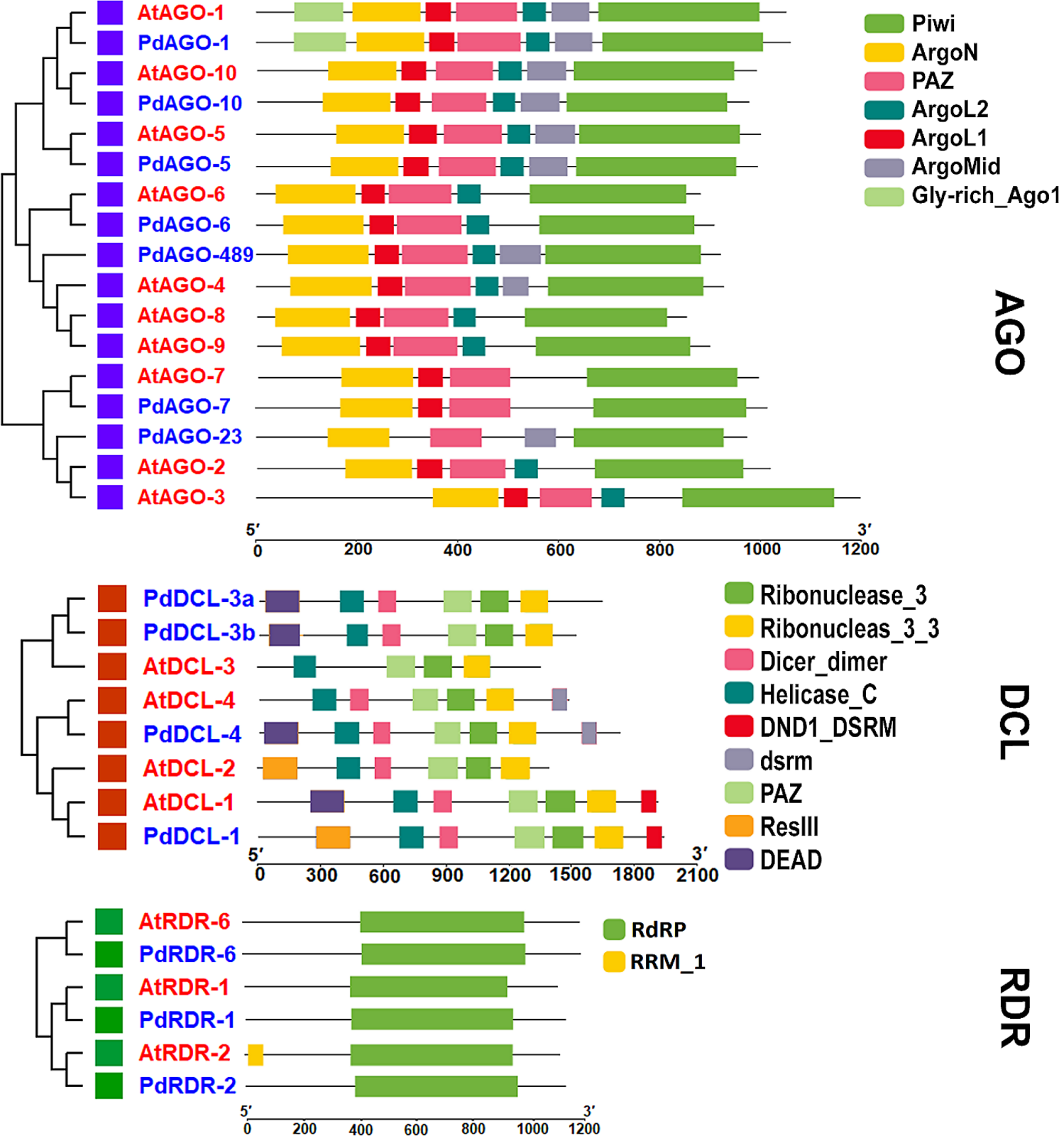
The conserved domains of AtRNAi and the PdRNAi proteins. Different color represents different conserved domains. Here Piwi indicates Piwi domain, ArgoN indicates N-terminal domain, PAZ indicates PAZ domain, ArgoL2 indicates Argonaute linker 2 domain, ArgoL1 indicates Argonaute linker 1 domain, ArgoMid indicates Mid domain of argonaute, Gly-rich_Ago1 indicates Glycine-rich region of Argonaut, RNase III indicates Ribonuclease III domain, Dicer_dimer indicates Dicer dimerization domain, Helicase_C indicates Helicase conserved C-terminal domain, dsrm indicates Double-stranded RNA binding motif, DEAD indicates DEAD/DEAH box helicase domain, RdRP indicates RNA dependent RNA polymerase, RRM_1 RNA recognition motif 1

In this study, we have compared the conserved domains of PdDCL, PdAGO, and PdRDR with the AtDCL, AtAGO, and AtRDR proteins. From Fig. 3, we have observed that both PdDCLs and AtDCLs shows almost similar conserved domains, including Helicase_C, Dicer_dimer, PAZ, RNase III, DND1-DSRM, DEAD, dsrm, ResIII domain. Both PdAGOs and AtAGOs have nearly identical conserved domains, such as Piwi, ArgoN, PAZ, ArgoL1, ArgoL2, ArgoMid, and Gly-rich_Ago1 domains. The Gly-rich_Ago1 domain has been found in PdAGO-1 and AtAGO-1. We have found that the PdRDRs and AtRDRs have almost the same conserved domains, including RNA-dependent RNA polymerase (RdRP) domain (Fig. 3). We have selected 10 significant motifs for PdRNAi and AtRNAi proteins by using MEME-suite analysis. Most of the PdDCL and AtDCL proteins have shown almost similar motif distributions. The annotated conserved motifs 1, 3, 4, 5, and 7 represents Ribonuclease 3 domain, motif 2 represents the Helicase-C domain, motif 6 represents the DEAD domain, motif 8 represents the Dicer dimer domain, and motif 10 represents the PAZ domain, of the predicted PdDCL proteins (Fig. S3A).

The annotated conserved motifs 1, 2, 3, 4, 6, 7, and 8 represent the Piwi domain, motif 5 represent the ArgoL1 domain, motif 10 represent the ArgoL2 domain and motif 9 represent the PAZ domain of predicted PdAGO proteins. The annotated conserved motifs 1–10 represent the RdRP domain of projected PdRDR proteins. The MEME studies of conserved motifs in the PdDCL, PdAGO, and PdRDR protein families have revealed comparable functional diversity in date palms. We have also compared the domains and motifs of PdRNAi proteins with OsRNAi proteins. We have perceived that PdDCLs, PdAGOs, and PdRDRs show similar domain and motif patterns according to OsDCLs, OsAGOs, and OsRDRs, respectively (Fig. S2 and S3B).

### PdRNAi genes structures with respect to AtRNAi genes

**Fig. 4:**
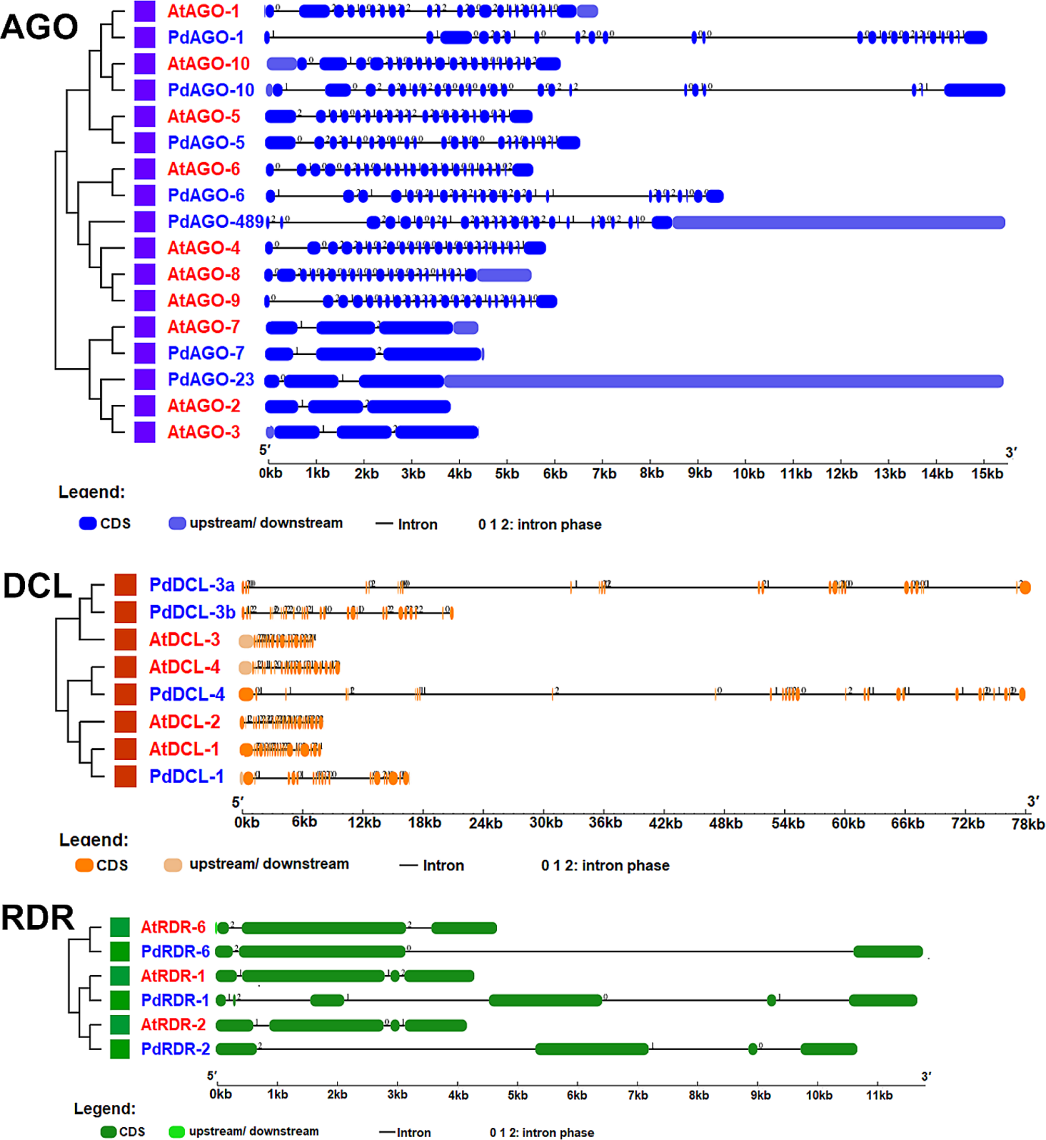
Structure of AtRNAi and the predicted PdRNAi genes

The exon-intron structures of PdRNAi genes have been analyzed with respect to the AtRNAi genes to investigate the similarities of exon-intron between them. We have observed that the exon-intron structures of *PdDCL*s, *PdAGO*s, and *PdRDR*s are almost identical with the *AtDCL*s, *AtAGO*s, and *AtRDR*s (Fig. 4). According to the gene structure analysis, the *PdDCL*s have 19–28 exons, *PdAGO*s have 22–26 exons and *PdRDR*s have 3–6 exons, which has been nearly identical to *AtDCL*s *AtAGO*s and *AtRDR*s, respectively. The gene *PdAGO-7* has three and *PdAGO-2,3* gene has four exons. We have also compared the similarities of exon-intron patterns between PdRNAi and OsRNAi genes and found their similarities like AtRNAi genes (Fig. S4).

### Sub-cellular localizations of PdRNAi proteins with respect to AtRNAi proteins

**Fig. 5:**
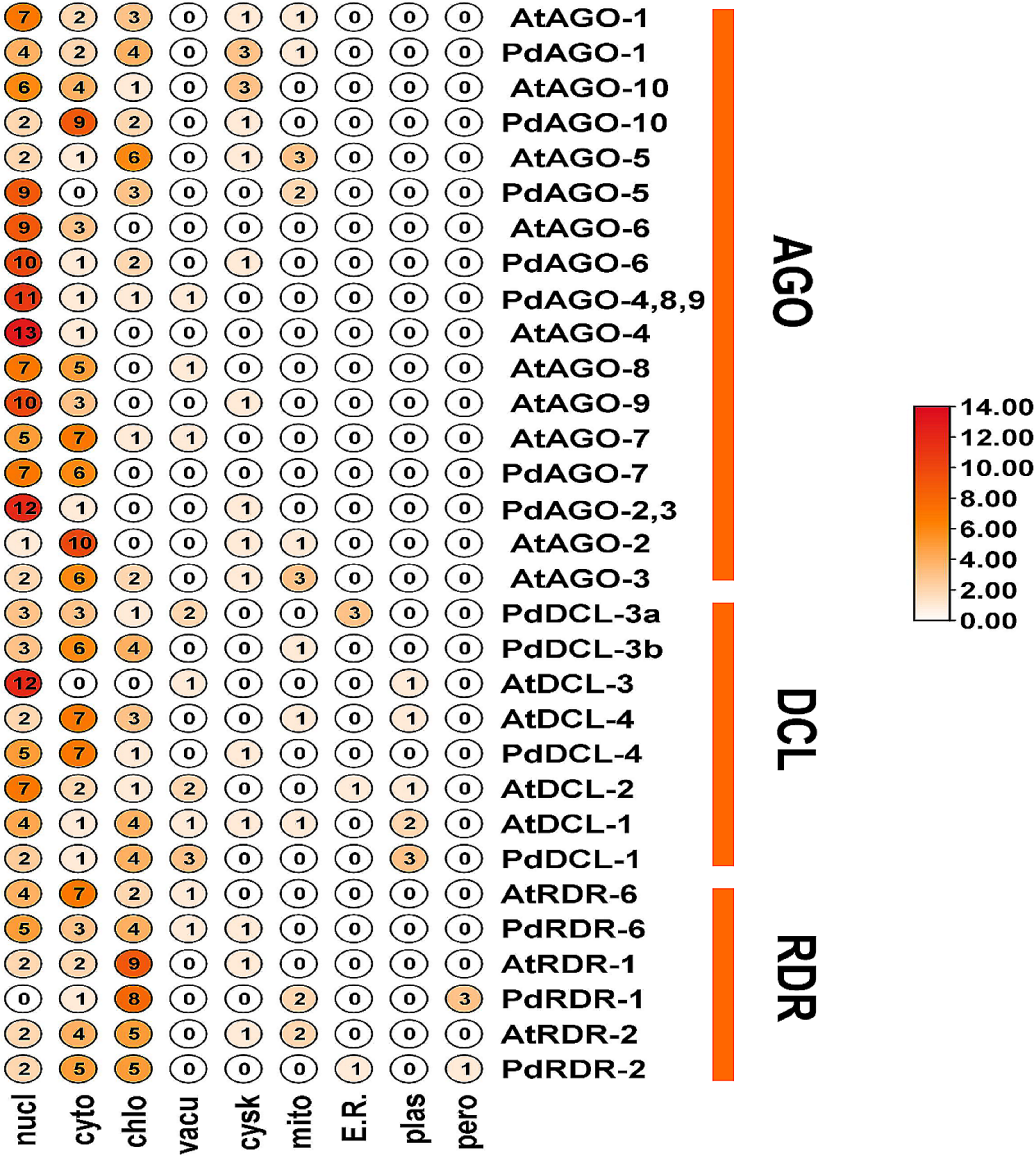
Sub-cellular localization analysis of AtRNAi and PdRNAi proteins. Protein percentages

Subcellular localization investigations has been carried out to learn more about the PdRNAi proteins cellular presence. The majority of PdRNAi, as well as AtRNAi proteins, have found in the nucleus, chloroplast, and cytosol, according to sub-cellular localization study (Fig. 5). Some of our PdRNAi proteins have also found in cell vacuoles, cytoskeletal and mitochondria (Fig. 5). In addition, We have also compared the sub-cellular locations between PdRNAi and OsRNAi proteins and noticed their similarities like AtRNAi proteins (Fig. S5).

are found in several biological components. In this analysis, nucl– nucleus, cyto– cytosol, chlo– chloroplast, vacu– vacuole, cysk– Cytoskeletal, mito– mitochondria, E.R.– Endoplasmic Reticulum, plas– plastid, pero– peroxisome.

### Biological processes and molecular functions of PdRNAi proteins

The GO enrichment analysis has been performed to see how predicted PdRNAi proteins are connected with BPs and MFs. The study have identified several important BPs and MFs those are associated with gene silencing processes (Table 2). For example, 9 (PdDCL-1, PdDCL-3b, PdDCL-4, PdAGO-2,3, PdAGO-4,8,9, PdAGO-7, PdAGO-10, PdRDR-1, PdRDR-6) of 14 PdRNAi proteins have been engaged with BP term response to virus (GO:0009615; *p*-value: 1.8E-21), eight (PdDCL-1, PdDCL-3b, PdDCL-4, PdAGO-4,8,9, PdAGO-7, PdRDR-1, PdRDR-2, PdRDR-6) of 14 PdRNAi proteins have been found to be involved in the BP terms as production of siRNA (GO:0030422; *p*-value: 6.5E-21), RNA interference (GO:0016246; *p*-value: 1.4E-20), dsRNA fragmentation (GO:0031050; *p*-value: 3.1E-19). Our study also found that, 4 (PdDCL-1, PdDCL-3b, PdAGO-4,8,9, PdRDR-2) of 14 PdRNAi proteins have been found to be involved in the CC terms as nuclear lumen (GO:0031981; *p*-value: 0.00019), intracellular organelle lumen (GO:0070013; *p*-value: 0.00025), organelle lumen (GO:0043233; *p*-value: 0.00025), membrane-enclosed lumen (GO:0031974; *p*-value: 0.00026). Three (PdRDR-1, PdRDR-2, PdRDR-6) of 14 PdRNAi proteins have been found to be involved in the molecular function term RNA-directed RNA polymerase activity (GO:0003968; *p*-value: 2E-08). Three (PdDCL-1, PdDCL-3b, PdDCL-4) of 14 PdRNAi proteins have been found to be involved in the molecular functions terms as ribonuclease III activity (GO:0004525; *p*-value: 3.1E-08), double-stranded RNA-specific ribonuclease activity (GO:0032296; *p*-value: 3.1E-08). Two (PdAGO-2,3, PdAGO-4,8,9) of 14 PdRNAi proteins have been found to be involved in the MF term siRNA binding (GO:0035197; *p*-value: 2.2E-06). We have also compared the GOs of PdRNAi with AtRNAi and OsRNAi proteins and perceived their similarities (Table 2).

**Table 2:** GO enrichment analysis results with the AtRNAi, predicted PdRNAi and OsRNAi proteins

GO.ID	Term	GO-terms	Enriched RNAi proteins
GO:0009615	response to virus	BP	PdAGO-2,3, PdDCL-3b, PdDCL-1, PdDCL-4, PdAGO-7, PdRDR-1, PdAGO-4,8,9, PdRDR-6, PdAGO-10
			AtRDR-1, AtAGO-2, AtAGO-3, AtAGO-1, AtAGO-7, AtAGO-4, AtAGO-5, AtAGO-6, AtDCL-2, AtDCL-3, AtRDR-6, AtDCL-4, AtAGO-9, AtAGO-10
			SHL2, OsAGO1a, OsRDR1, OsDCL1a, SHL4, OsDCL2a, OsMEL1, OsAGO4b, SHO1, OsAGO2, OsPNH1, OsDCL3b
GO:0030422	production of siRNA involved in RNA interference	BP	PdDCL-3b, PdDCL-1, PdDCL-4, PdRDR-2, PdAGO-7, PdRDR-1, PdAGO-4,8,9, PdRDR-6
			AtDCL-1, AtRDR-1, AtAGO-7, AtAGO-4, AtAGO-6, AtDCL-2, AtDCL-3, AtRDR-6, AtRDR-2, AtDCL-4
			SHL2, OsRDR1, OsDCL1a, SHL4, OsDCL2a, OsAGO4b, OsRDR2, SHO1, OsDCL1c, OsDCL1b, OsDCL2b, OsDCL3b
GO:0016246	RNA interference	BP	PdDCL-3b, PdDCL-1, PdDCL-4, PdRDR-2, PdAGO-7, PdRDR-1, PdAGO-4,8,9, PdRDR-6
			AtDCL-1, AtRDR-1, AtAGO-7, AtAGO-4, AtAGO-6, AtDCL-2, AtDCL-3, AtRDR-6, AtRDR-2, AtDCL-4,
			SHL2, OsRDR1, OsDCL1a, SHL4, OsDCL2a, OsAGO4b, OsRDR2, SHO1, OsDCL1c, OsDCL1b, OsDCL2b
GO:0031050	dsRNA fragmentation	BP	PdDCL-3b, PdDCL-1, PdDCL-4, PdRDR-2, PdAGO-7, PdRDR-1, PdAGO-4,8,9, PdRDR-6
			AtDCL-1, AtRDR-1, AtAGO-7, AtAGO-4, AtAGO-6, AtDCL-2, AtDCL-3, AtRDR-6, AtRDR-2, AtDCL-4
			SHL2, OsRDR1, OsDCL1a, SHL4, OsDCL2a, OsAGO4b, OsRDR2, SHO1, OsDCL1c, OsDCL1b, OsDCL2b, OsDCL3b
GO:0003968	RNA-directed RNA polymerase activity	MF	PdRDR-2, PdRDR-1, PdRDR-6
			AtRDR-1, AtRDR-6, AtRDR-2
			OsRDR3, OsRDR4, SHL2, OsRDR1, OsRDR2
GO:0004525	ribonuclease III activity	MF	PdDCL-3b, PdDCL-1, PdDCL-4
			AtDCL-1, AtDCL-2, AtDCL-3, AtDCL-4
			OsDCL3a, OsDCL1a, OsDCL2a, SHO1, OsDCL1c, OsDCL1b, OsDCL2b, OsDCL3b
GO:0032296	double-stranded RNA-specific ribonuclease activity	MF	PdDCL-3b, PdDCL-1, PdDCL-4
			AtDCL-1, AtDCL-2, AtDCL-3, AtDCL-4
			OsDCL3a, OsDCL1a, OsDCL2a, SHO1, OsDCL1c, OsDCL1b, OsDCL2b, OsDCL3b
GO:0035197	siRNA binding	MF	PdAGO-2,3, PdAGO-4,8,9,
			AtAGO-2, AtAGO-1, AtAGO-4, AtAGO-6, AtAGO-9
			OsAGO1a, OsMEL1, OsAGO4b, OsAGO2

Here, GO.ID: gene ontology ID, BP: biological processes, and MF: molecular function

### PdRNAi gene regulatory network analysis

#### *Trans*-regulatory factors of PdRNAi genes

**Fig. 6:**
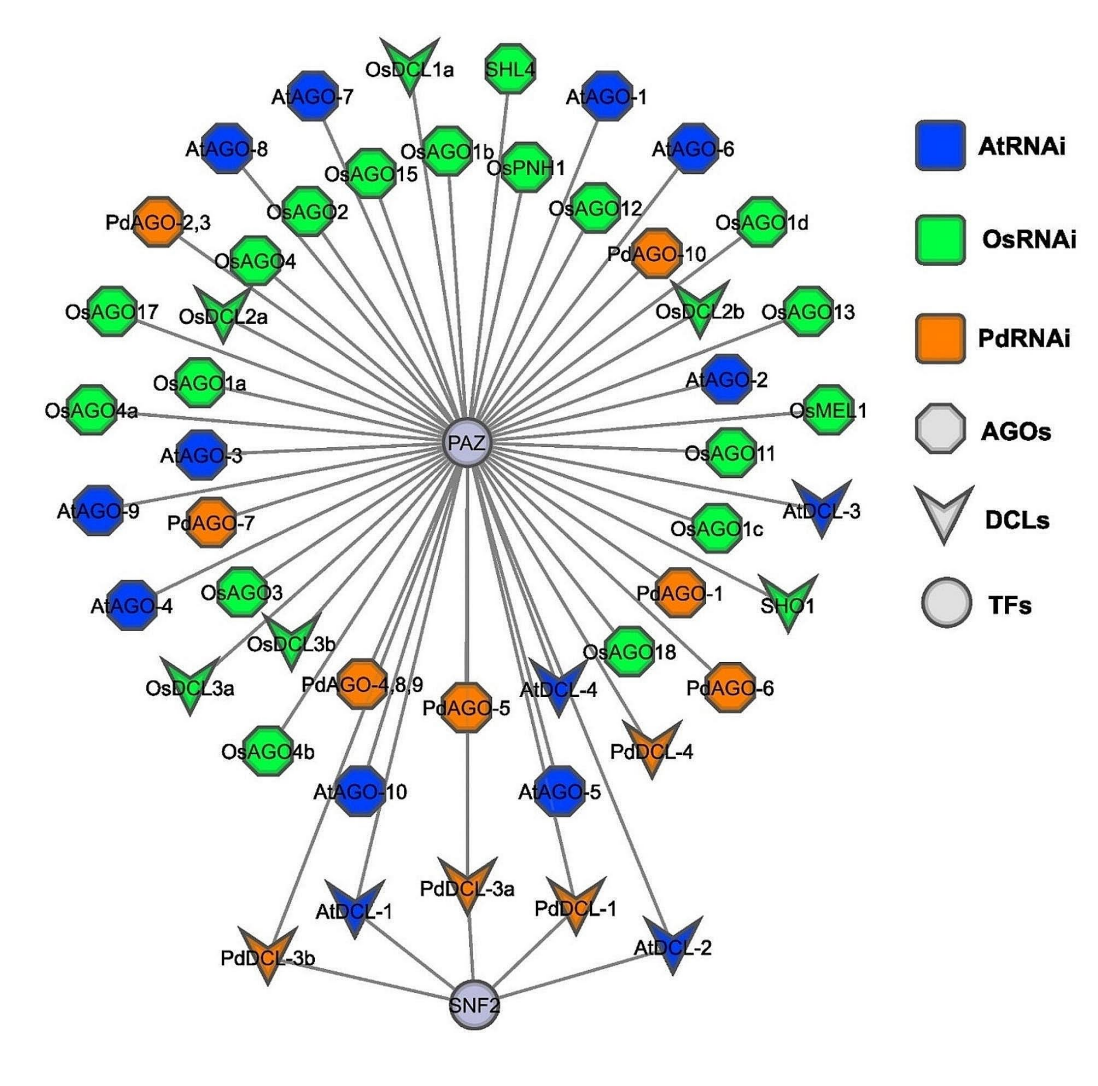
The regulatory network among the TFs with PdRNAi, AtRNAi, And OsRNAi genes. The nodes of the network have been colored based on RNAi genes: AtRNAi: blue, OsRNAi: light green, and PdRNAi: orange. The node shape of the network have been based on RNAi genes: DCLs: triangle, AGOs: octagonal, and TFs: round

At first, we have identified transcription factors (TFs) with respect to AtRNAi, PdRNAi, and OsRNAi genes by using PlantTFcat web-tool. Then we have submitted AtRNAi, PdRNAi, and OsRNAi genes and associated TFs in Cytoscape to construct the network between them. We have found two (PAZ, SNF2) TFs in AtRNAi, PdRNAi, and OsRNAi genes (Fig. 6). The PAZ TF has been found to associated with (*PdDCL-1, PdAGO-7, PdAGO-10, PdDCL-4, PdDCL-3a, PdDCL-3b, PdAGO-1, PdAGO-2,3, PdAGO-4,8,9, PdAGO-5, PdAGO-6, AtDCL-1, AtAGO-6, AtAGO-7, AtAGO-8, AtAGO-9, AtAGO-10, AtDCL-2, AtDCL-3, AtDCL-4, AtAGO-1, AtAGO-2, AtAGO-3, AtAGO-4, AtAGO-5, OsAGO1a, OsMEL1, OsAGO13, SHL4, OsPNH1, OsAGO17, OsAGO12, OsAGO11, OsAGO18, OsAGO15, OsAGO1b, OsDCL1a, OsDCL2a, OsDCL2b, OsDCL3a, OsDCL3b, SHO1, OsAGO1c, OsAGO1d, OsAGO2, OsAGO3, OsAGO4a, OsAGO4b, OsAGO4*) AtRNAi, PdRNAi, and OsRNAi genes. The SNF2 associated with *AtDCL-1, AtDCL-2, PdDCL-1, PdDCL-3a, and PdDCL-3b* genes (Fig. 6).

### *Cis*-regulatory factors of PdRNAi genes

**Fig. 7:**
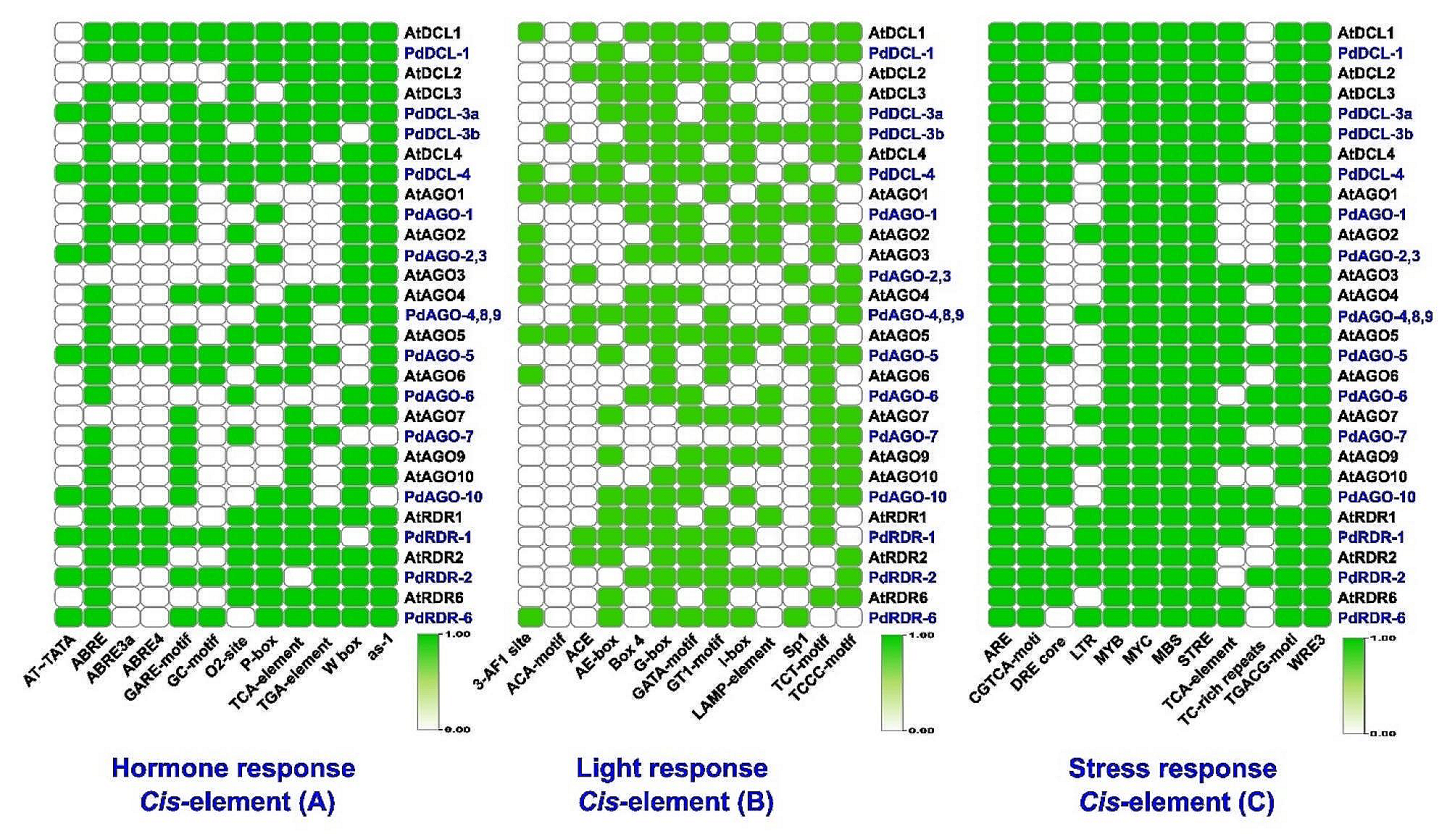
The *cis*-acting regulatory elements of AtRNAi and predicted PdRNAi genes. The deep to light green color represent the presence degree of that element with the corresponding genes. (**A**) hormone-responsive *cis*-elements, (**B**) light-responsive *cis*-elements, and (**C**) stress-responsive *cis*-elements

The *cis*-acting regulatory elements (CAREs) study has been performed to investigate how those regulators controlled PdRNAi genes. According to our findings, the regulatory regions of PdRNAi genes have contained light-responsive (LR), hormone-responsive (HR), and stress-responsive (SR) related motifs (Fig. 7). The LR motifs including 3-AF1 binding site, ACE (ACGT-containing element), AE-box (activation element), Box 4 (involved in light responsiveness), G-box (CACGTG motif), GATA-motif, GT1-motif (GGTTAA, GGTAATT, GGTAAAT, GTTAC, TACAGT, and GGTAAA), I-box (AGATATGATAAAA), LAMP-element, Sp1 (specificity protein 1), TCT-motif (polypyrimidine initiator), and TCCC-motif have been found in PdRNAi genes (Fig. 7). The significant number of I-box LR motif has found in the expected PdRNAi genes. The *cis*-acting regulatory elements ABRE (involved in the abscisic acid responsiveness), O2-site (involved in zein metabolism regulation), P-box (gibberellin-responsive element), TCA-element (involved in salicylic acid responsiveness), and TGA-element (auxin-responsive element) has found in the promoter regions of PdRNAi genes, that are involved in the regulation of hormones (HR). Some of our PdRNAi genes are regulated by the *cis*-acting elements, including TC-rich repeats (involved in defense and stress responsiveness, MBS (*MYB* binding site involved in drought-inducibility), *MYB* (involved in drought-inducibility, LTR (involved in low-temperature responsiveness as stress-responsive (SR). We have also compared the *cis*-elements between PdRNAi and OsRNAi genes and observed their similarities like AtRNAi genes (Fig. S6).

### miRNA of PdRNAi genes

The purpose of the miRNA investigation is to see how miRNA regulates the expression of PdRNAi genes. We have detected eight key miRNAs, including Pda-miR156b, Pda-miR157b, Pda-miR159a, Pda-miR166a, Pda-miR167d, Pda-miR395c, Pda-miR396a, Pda-miR529a, Pda-miR482, Pda-miR2118 using PdRNAi genes (Figs. S7A, B). Six of the 14 PdRNAi genes have been regulated by the Pda-miR529a. Four of the 14 PdRNAi genes have been regulated by the Pda-miR396a. The *PdDCL-1* has been regulated by Pda-miR166a, Pda-miR167d, Pda-miR396a, Pda-miR482, Pda-miR2118. The *PdDCL-4* has been regulated by Pda-miR156b, Pda-miR159a, Pda- miR395c, Pda-miR396a. All of the miRNAs are found to expressed in the leaves and roots. Both Pda-miR166a and Pda-miR2118 have a high expression score in leaves and roots. Both Pda-miR156b and Pda-miR159a have a medium expression score in leaf. In root, the expression scores of Pda-miR156b, Pda-miR159a, Pda-miR396a, Pda-miR482 are medium. We have also studied the miRNAs and expressions of OsRNAi and AtRNAi genes to compare with PdRNAi genes. We have found that OsRNAi and AtRNAi have almost similar miRNAs and miRNA expressions according to PdRNAi genes (Figs. S7A, B).

## Discussion

The major RNAi genes *RDR*, *DCL*, and *AGO* function as unique short RNAs that regulate a variety of biological processes and pathways by silencing protein-coding genes [80, 81]. In this study, we have identified 4 PdDCLs, 7 PdAGOs, and 3 PdRDRs as RNAi proteins from the date palm genome guided by AtRNAi proteins as a query sequence in BLASTp search based on MSAs. Then phylogenetic tree analysis has been performed to investigate the similarities of PdRNAi with AtRNAi and OsRNAi proteins. To validate the predicted PdRNAi proteins computationally, we have studied their characteristics and activities with respect to both AtRNAi and OsRNAi proteins. Here we considered OsRNAi proteins, since both of rice (*Oryza sativa*) and date palm belong to the same monocot commelinids clade (Fig. 2 and Fig. S1) [19]. In order to investigate the roles of PdRNAi proteins in the protein coding gene silencing, stress response and disease resistance, we have analyzed domains, structures, locations, molecular functions and regulatory factors of PdRNAi genes that are discussed below.

The domains and motifs of predicted PdRNAi proteins have displayed similar patterns compared with AtRNAi and OsRNAi proteins (Figs. 3 and 4 and Figs. S2, S3). All PdDCLs and three PdAGO proteins might be functions as protein coding gene silencers, since all PdDCLs and three PdAGOs belong to the same group of AtDCLs and AtAGOs (AtAGO-3, AtAGO-4, AtAGO-6, and AtAGO-9) protein [82–88]. The PdDCL-1 protein may produce 21 and 22nt sRNAs from small stem-loop RNA substrates, whereas PdDCL-2, PdDCL-3, and PdDCL-4 proteins may produce 22nt, 24nt, and 21nt sRNAs from large dsRNA, respectively [89]. The PAZ and RNase III domains in Dicer (PdDCLs) synthesize silencing-associated sRNAs from a larger dsRNA [12, 90]. The RDR (PdRDRs) synthesize the majority of long dsRNAs in plants [91]. The PdRDR-6 protein may be required for the production of trans-acting siRNAs (tasiRNAs) because it cleavages the dsRNA. The PdDCL-4 protein may synthesize the dsRNA from the miRNA-cleavage end, resulting in 21-nucleotide siRNA duplexes formation [92–94]. More specifically, the identified PIWI domain contains structural similarities with ribonuclease-H enzymes, which indicates that it may be responsible for cleaving the target mRNA [95]. The predicted DEAD-box and helicase domain may stimulate date palm to produce secondary siRNAs [96, 97]. The anticipated PAZ and RNase III domains may operate as an indication, creating miRNAs with comparable sizes [98]. The predicted PIWI and PAZ may recognize and cleave the target mRNA [99–101]. Most of the predicted PdRNAi proteins have been significantly enriched in the nucleus, cytosol, and chloroplast (Fig. 5 and Fig. S5), where the first two locations are known to be involved with the occurrence of the RNA silencing mechanism [102, 103]. The GO enrichment analysis of PdRNAi genes has detected some vital BP and MF terms that are directly involved with the gene silencing mechanisms and those terms are almost identical with AtRNAi and OsRNAi genes (Table 2). Out of 14 predicted PdRNAi proteins, 8 proteins are involved with the biological process terms as production of siRNA (GO:0030422) [104], RNA interference (GO:0016246) [105], and dsRNA fragmentation (GO:0031050) [106]. The GO:0030422 term is associated with the 24nt hc-siRNA and 21nt siRNA production pathways [107]. Eukaryotic cells use siRNAs (GO:0030422, GO:0035197) [108–111] to bind and enhance the degradation of specific endogenous mRNAs generated by dsRNA (GO:0031050. GO:0032296) [112–114], to decrease protein synthesis at the post-transcriptional stage. The PdRDR proteins are involved with the molecular function term RNA-directed RNA polymerase activity (GO:0003968) [115], which is associated with RNA-mediated gene silencing in plants [116]. Three PdDCL proteins are involved with the terms of the molecular function as ribonuclease III activity (GO:0004525) [117–119] which specifically cleave dsRNAs for RNAi mechanisms in plants [90]. Two PdRNAi proteins are involved with the molecular function term siRNA binding (GO:0035197), which is essential to the RNAi mechanisms [111]. TFs are key regulatory factors that are involved in the activation or deactivation of upstream signaling pathways and bind to specific promoter regions (i.e., *cis*-acting elements) of their target genes to increase or inhibit the transcriptional rate of those genes [120]. Our detected PAZ-Argonaute [121] (similar as OsRNAi) genes, a member of the PTGS family of TFs, may require for RNAi pathway (Fig. 6). The SNF2 is linked with three PdRNAi genes. The SNF2 protein may supply ATP-driven motor components for remodeling systems that may control gene silencing of PdRNAi genes [122]. The miRNAs function as critical regulators of eukaryotic gene expression by targeting mRNAs for break-down or translation inhibition [123]. PdRNAi and OsRNAi almost similar miRNAs (Figs. S7A, B). Our detected miRNAs have a variety of significant activities, including gene silencing (miR159a, miR482) [124, 125]. Thus the predicted PdRNAi genes through sRNAs may play a vital role for the protein coding genes silencing.

We found that two *PdAGO* genes, which belong to the gene groups of *AtAGO-10* and *AtAGO-7* genes and are associated with *A. thaliana* development, may the best candidates for date palm development [126, 127]. The PdRNAi belong to the gene groups of *OsDCL-4, OsAGO-1* (a-d), and *SHL2*, which may enhance the expressions of target genes during early floral/panicle development [19]. The *PdAGO-5* gene, like *OsMEL-1* and *OsAGO-4* genes, might be expressed in reproductive organs and influence panicle and seed development. Similar to *OsPNH-1*, the *PdAGO-10* gene may influence SAM and leaf development [19]. The *PdRDR-2*, similar to *OsRDR-2*, may affect flower development [19]. The *PdDCL1, PdAGO1, PdAGO4*, and *PdRDR5* genes may play essential roles in stress response [128]. The PdDCLs might stimulate leaf or stem development in date palm as the intron number of each of PdDCLs and OsDCLs is almost the same as that of AtDCLs [28, 81, 90, 129–133] (Fig. 4 and Fig. S4) [134]. We have also found our detected *cis*-regulatory elements in OsRNAi (Fig. 7 and Fig. S6). Previous research has found that our predicted LR-related motifs play an important role in the photosynthetic process of leaves [9, 135–138]. The *cis*-acting regulatory elements ABRE [139, 140], O2-site [141], P-box, TCA-element, and TGA-element [141–143] have been discovered in the promoter regions of PdRNAi genes, that are involved in the regulation of hormones (HR). The TCA-elements, MYC, ABRE, and 3-AF1 binding sites are significant *cis*-regulatory elements of PdRNAi genes that might be played a vital role against plant’s bio/abiotic stress [144]. The regulatory effect of MYC on miRNA expression can be either positive or negative [145, 146]. Some of our PdRNAi genes are regulated as stress-responsive (SR) by the *cis*-acting elements, including TC-rich repeats [147], MBS, *MYB* [148], LTR [149]. Some of PdRNAi genes are influenced by the *cis*-acting factors, including Box-4, G-box, I-box, GT1 motif, GATA-motif, and TCT-motif that plays an important role as light response (LR) of several species [135–138, 150–154]. Because of LR-related motifs are important in photosynthetic mechanisms in plant leaves, the PdRNAi genes might be played an important role in improvement of date palms [155]. Our detected miRNAs have a variety of significant activities, including plant improvement (miR156b, miR157b, and miR396a) [156–158] and stress tolerance (miR166a, miR395e, miR529a) [159–161]. Thus, the predicted PdRNAi might be improved the quality of date palm fruits by promoting photosynthetic activity, a shorter flowering period, meristem, leaf, seed improvement and plant enhancement against different biotic and abiotic stresses.

We found that all *PdDCLs* and three *PdAGOs* belong to the same gene groups of *AtDCLs* and *AtAGOs*, which have anti-pathogen activity in *Arabidopsis* and may protect *P. dactylifera* from pathogens [40, 162–166]. The Ribonuclease III (found in PdDCLs) can resist pathogens [167] and the PdRDR proteins may have antiviral activity [168]. Date palm antiviral [169] and antifungal [170] defense may rely heavily on RdRP (GO:0003968) activity [171]. The dsRNA-specific ribonuclease activity (GO:0032296) [172] may target several date palm RNA viruses [173]. Our detected miRNAs in PdRNAi genes may have a variety of significant activities, including resistance to disease (miR167d, miR2118) [174, 175]. Thus, our predicted PdRNAi genes might be enhanced the date palm growth and development by regulating the respective protein coding genes against disease risks.

## Conclusion

In this study, we have identified 4 *PdDCL*, 7 *PdAGO*, and 3 *PdRDR* genes as PdRNAi genes guided by AtRNAi genes through bioinformatics analysis. To gain a better knowledge of PdRNAi corresponding to AtRNAi and OsRNAi gene families, we have investigated their phylogenetic relationship, domain components, genomic structure, subcellular localization, functional annotations, TFs, *cis*-elements, and miRNAs. The analysis results of this study have indicated that the *PdAGO-5, PdAGO-7*, and *PdAGO-10* genes might be highly involved in growth and development, and *PdDCL1, PdAGO1, PdAGO4* and *PdRDR5* might be effective against different stresses in date palm. So far, this is the first study to shed light on the major RNAi gene families of date palms. Therefore, the knowledge gathered from this study might be useful resources to enhanced the date palm production by regulating the respective protein coding genes against biotic and abiotic stresses. However, wet-lab experiments are needed to confirm that PdRNAi genes regulate date palm growth and development through the silencing mechanism of the releted protein-coding genes under various stresses.

### Electronic supplementary material

Below is the link to the electronic supplementary material.

Supplementary Material 1

## Data Availability

The dataset on date palm genome analyzed in this study has downloaded from the NCBI database with GenBank Accession Number: GCA_009389715.1 (NCBI taxonomy ID: 42345, BioSample:SAMN05011615, https://www.ncbi.nlm.nih.gov/genome/?term=Phoenix+dactylifera) using the published date palm genome sequence. The rest of the data that have used in this study will be available on request.
