# Supplementary material for: Genome-wide identification and in silico characterization of major RNAi gene families in date palm (Phoenix dactylifera)
Source: BMC Genom Data. 2024 Mar 15;25:31. doi: 10.1186/s12863-024-01217-x (PMC10943882; doi:10.1186/s12863-024-01217-x)
Supplement: Supplementary file 1 — Supplementary Material 1 [file 12863_2024_1217_MOESM1_ESM.pdf]

Genome-wide identification and *in silico* characterization of major RNAi gene families in Date palm (*Phoenix dactylifera*)

Md. Darun Naim[1], Md. Asif Ahsan[1], Ahmed Imtiaj[2] & Md. Nurul Haque Mollah[1*]

[1]Bioinformatics Lab, Department of Statistics, Faculty of Science, University of Rajshahi, Rajshahi-6205, Bangladesh
[2]Department of Botany, Faculty of Biological Sciences, University of Rajshahi, Rajshahi-6205, Bangladesh.

***Corresponding Author:** Md. Nurul Haque Mollah (mollah.stat.bio@ru.ac.bd)

(Supplementary Figures)

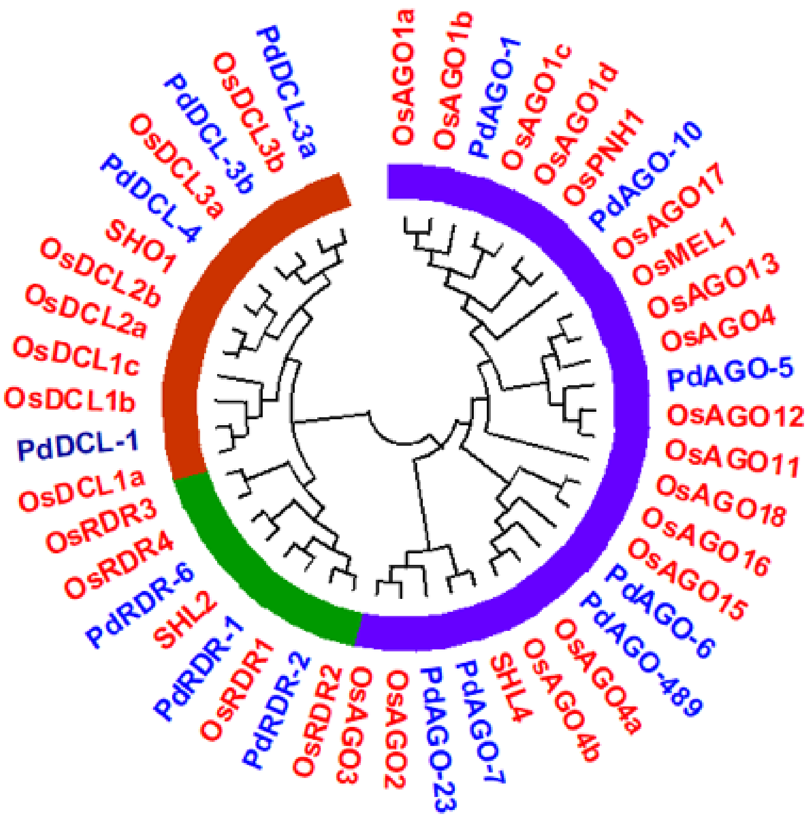

Fig. S1: The combined phylogenetic tree. In this tree, the PdRNAi (PdDCLs, PdAGOs, PdRDRs) and OsRNAi (OsDCLs, OsAGOs, OsRDRs) proteins are represented by green and red, respectively. Here AGOs, DCLs and RDRs families are represented by purple, orange and green circles, respectively.

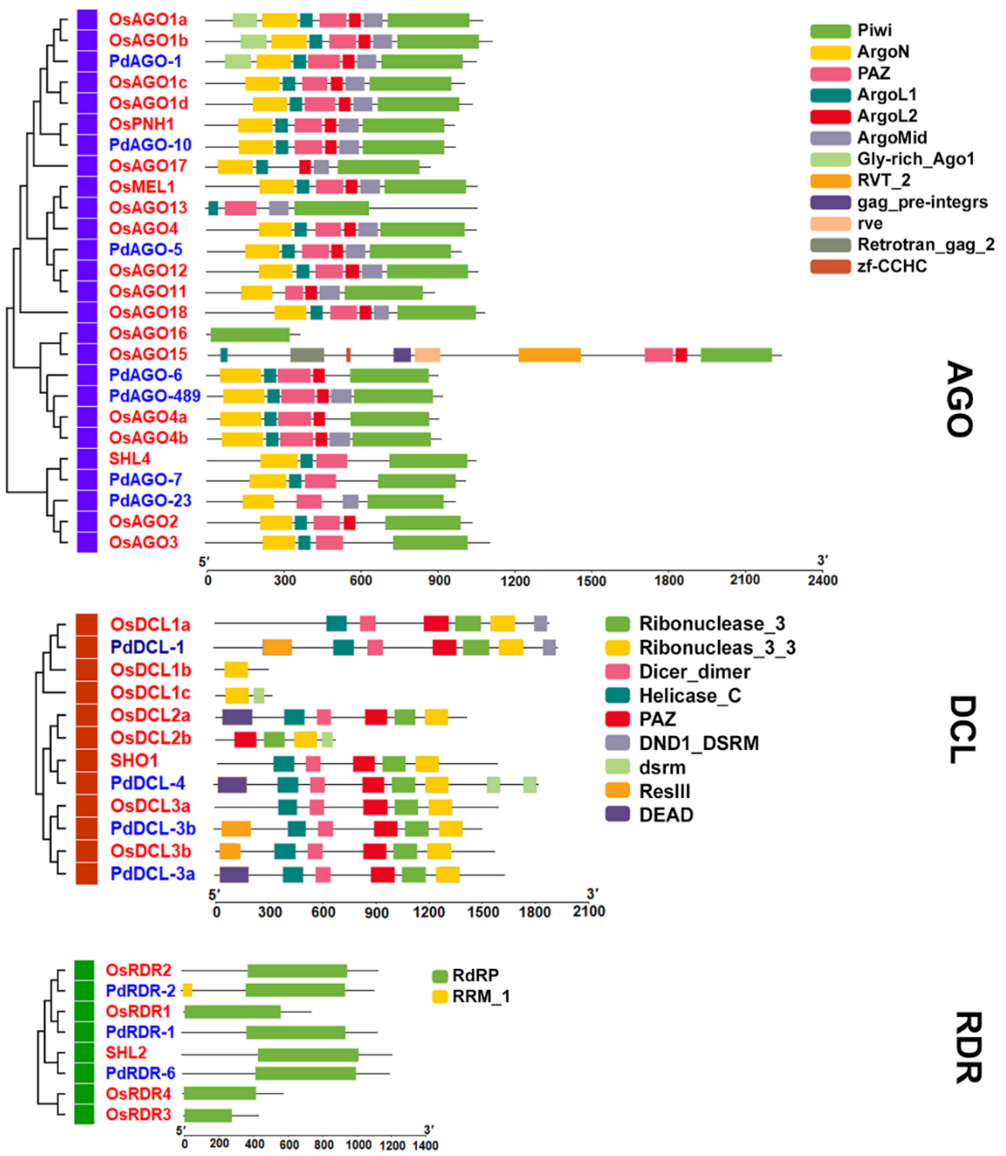

Fig. S2: The conserved domains of OsRNAi and the PdRNAi proteins. Different color represents different conserved domains. Here Piwi indicates Piwi domain, ArgoN indicates N-terminal domain, PAZ indicates PAZ domain, ArgoL2 indicates Argonaute linker 2 domain, ArgoL1 indicates Argonaute linker 1 domain, ArgoMid indicates Mid domain of argonaute, Gly-rich_Ago1 indicates Glycine-rich region of Argonaut, RNase III indicates Ribonuclease III domain, Dicer_dimer indicates Dicer dimerization domain, Helicase_C indicates Helicase conserved C-terminal domain, dsrm indicates Double-stranded RNA binding motif, DEAD indicates DEAD/DEAH box helicase domain, RdRP indicates RNA dependent RNA polymerase, RRM_1 RNA recognition motif 1.

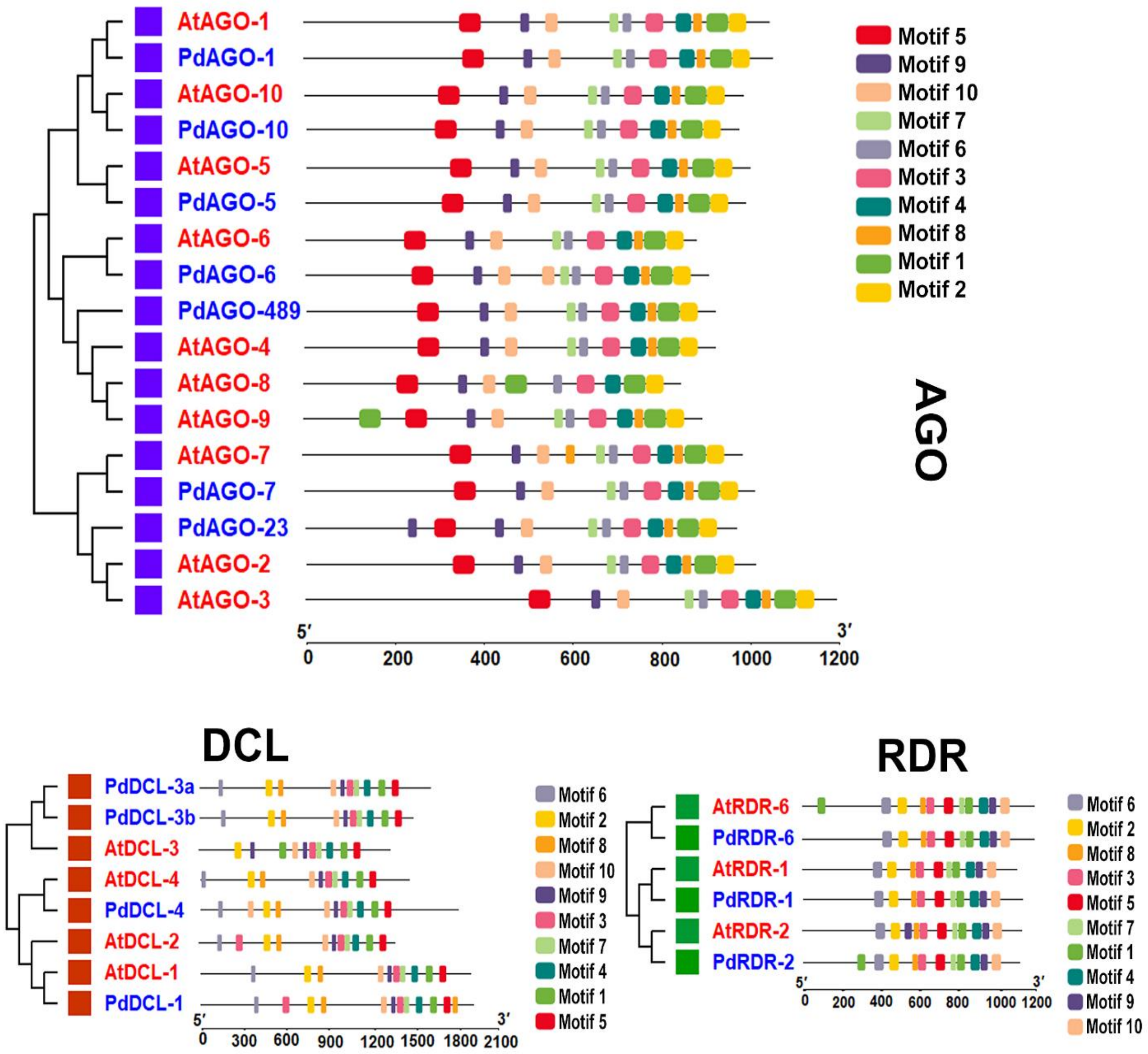

Fig. S3A: Motifs of AtRNAi and the PdRNAi proteins. Different color represents different motifs.

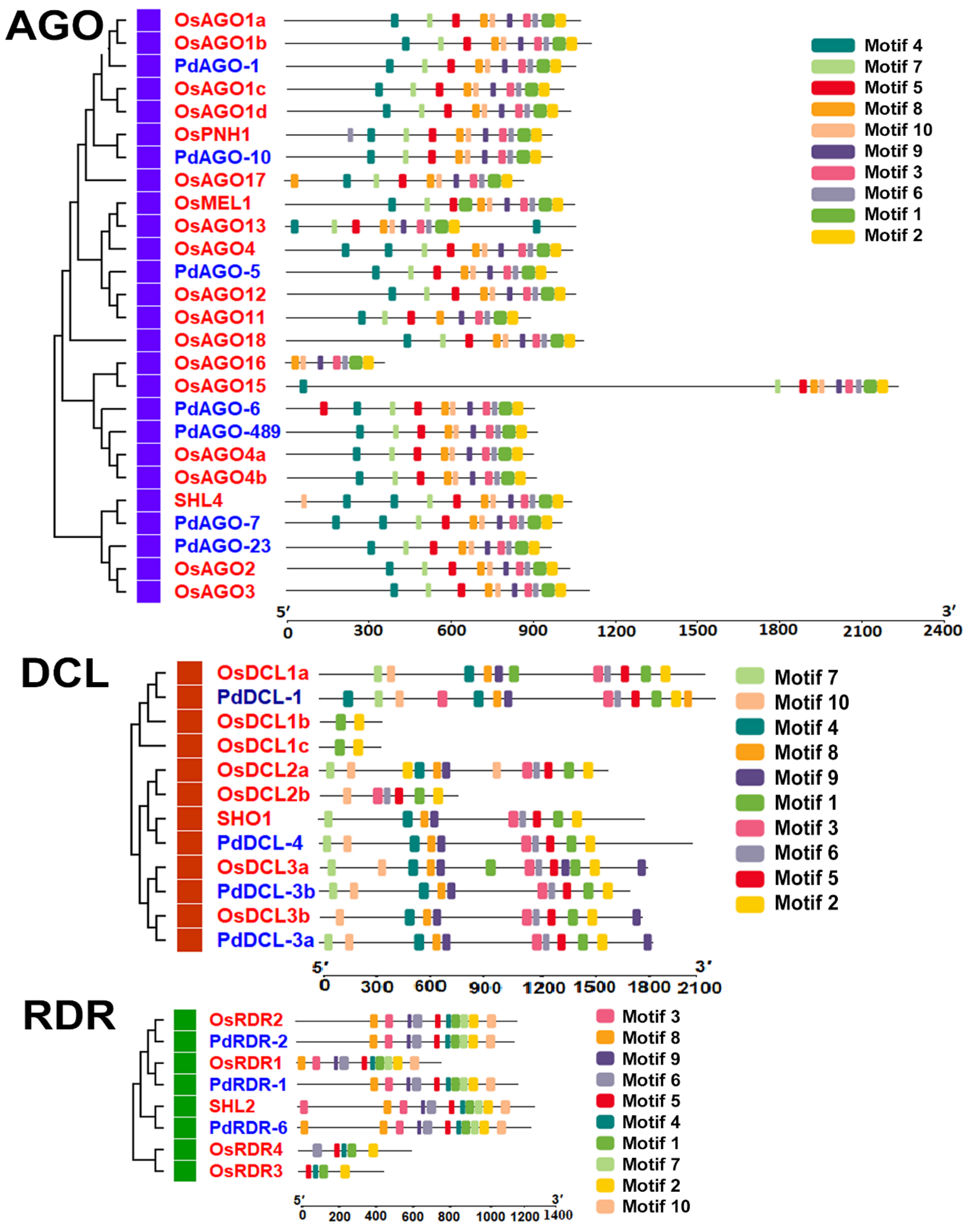

Fig. S3B: Motifs of OsRNAi and the PdRNAi proteins. Different color represents different motifs.

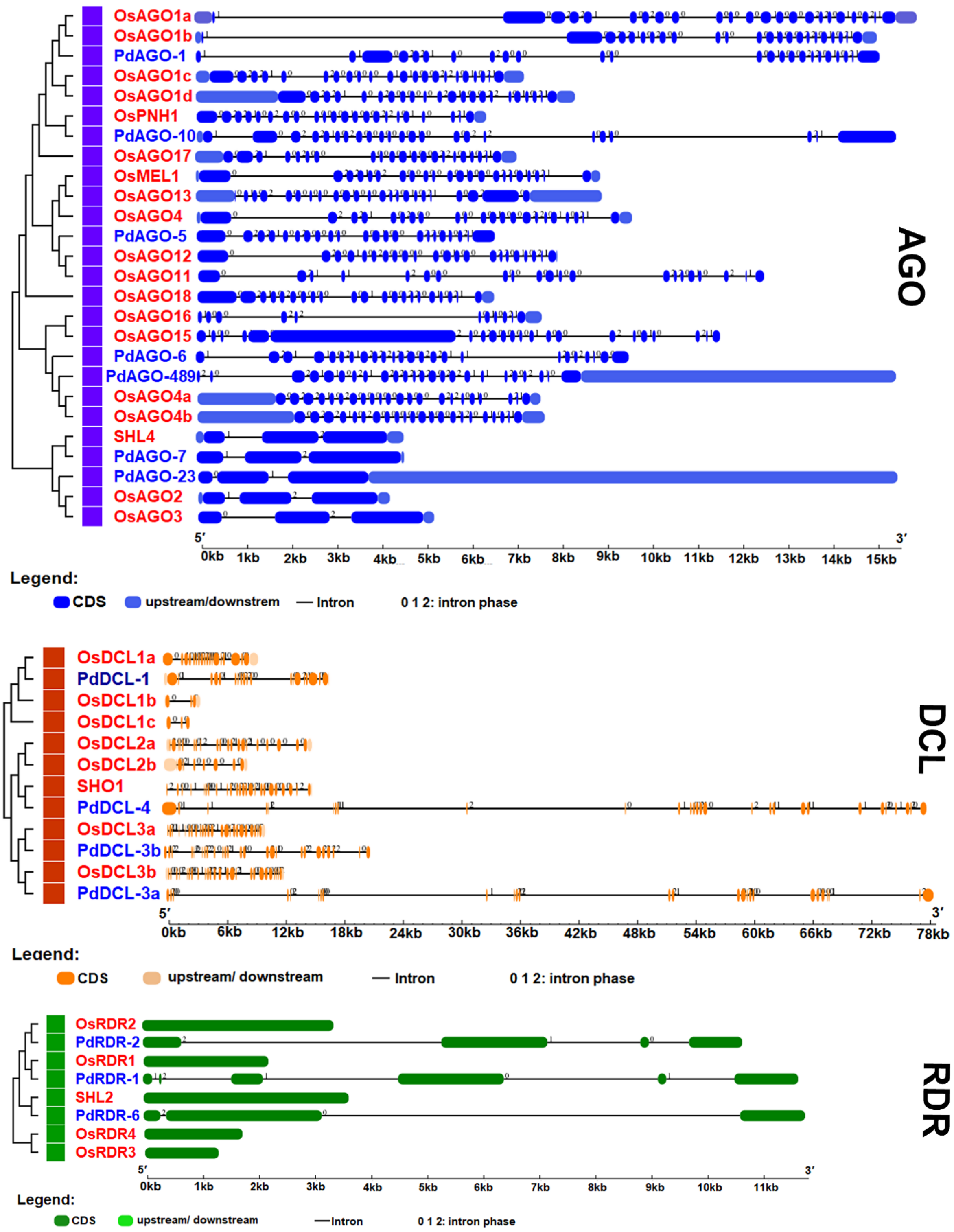

Fig. S4: Structure of OsRNAi and the predicted PdRNAi genes.

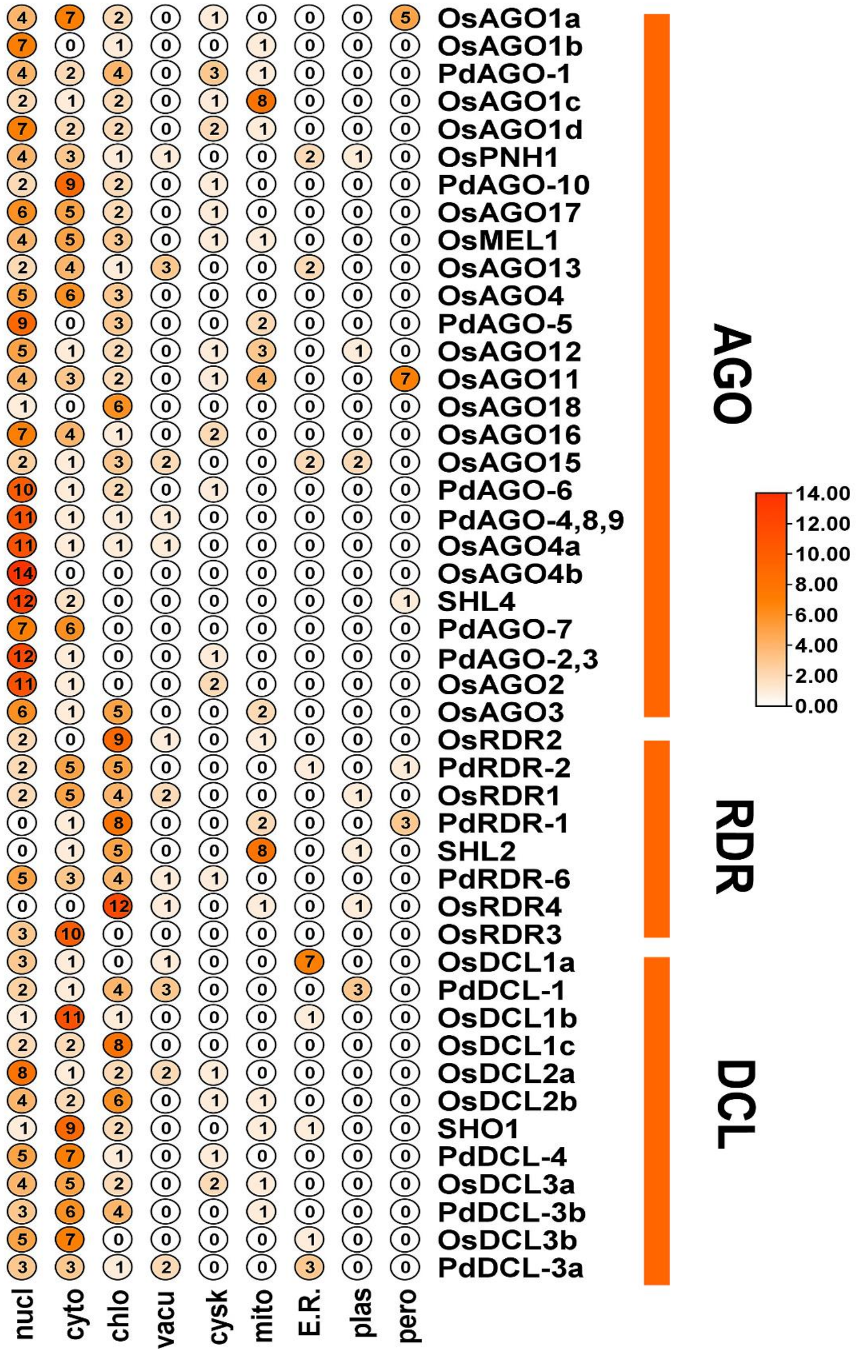

Fig. S5: Sub-cellular localization analysis of OsRNAi and PdRNAi proteins. Protein percentages are found in several biological components. In this analysis, nucl – nucleus, cyto – cytosol, chlo – chloroplast, vacu – vacuole, cysk – Cytoskeletal, mito – mitochondria, E.R. – Endoplasmic Reticulum, plas – plastid, pero – peroxisome.

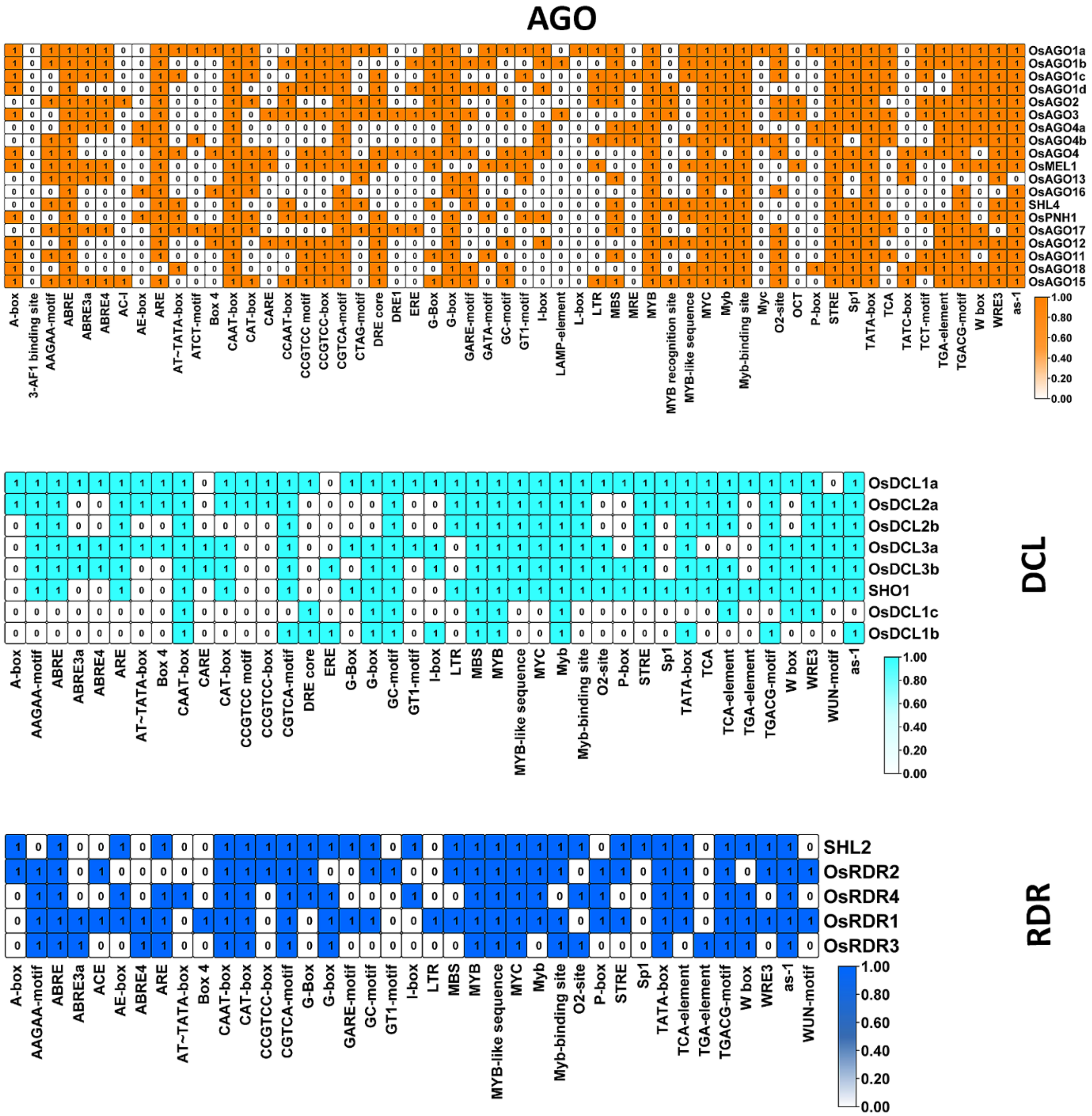

Fig. S6: The *cis*-acting regulatory elements in the upstream promoter region of OsDCL, OsAGO and OsRDR genes, respectively. The deep to light color represents the presence of that element with the corresponding genes.

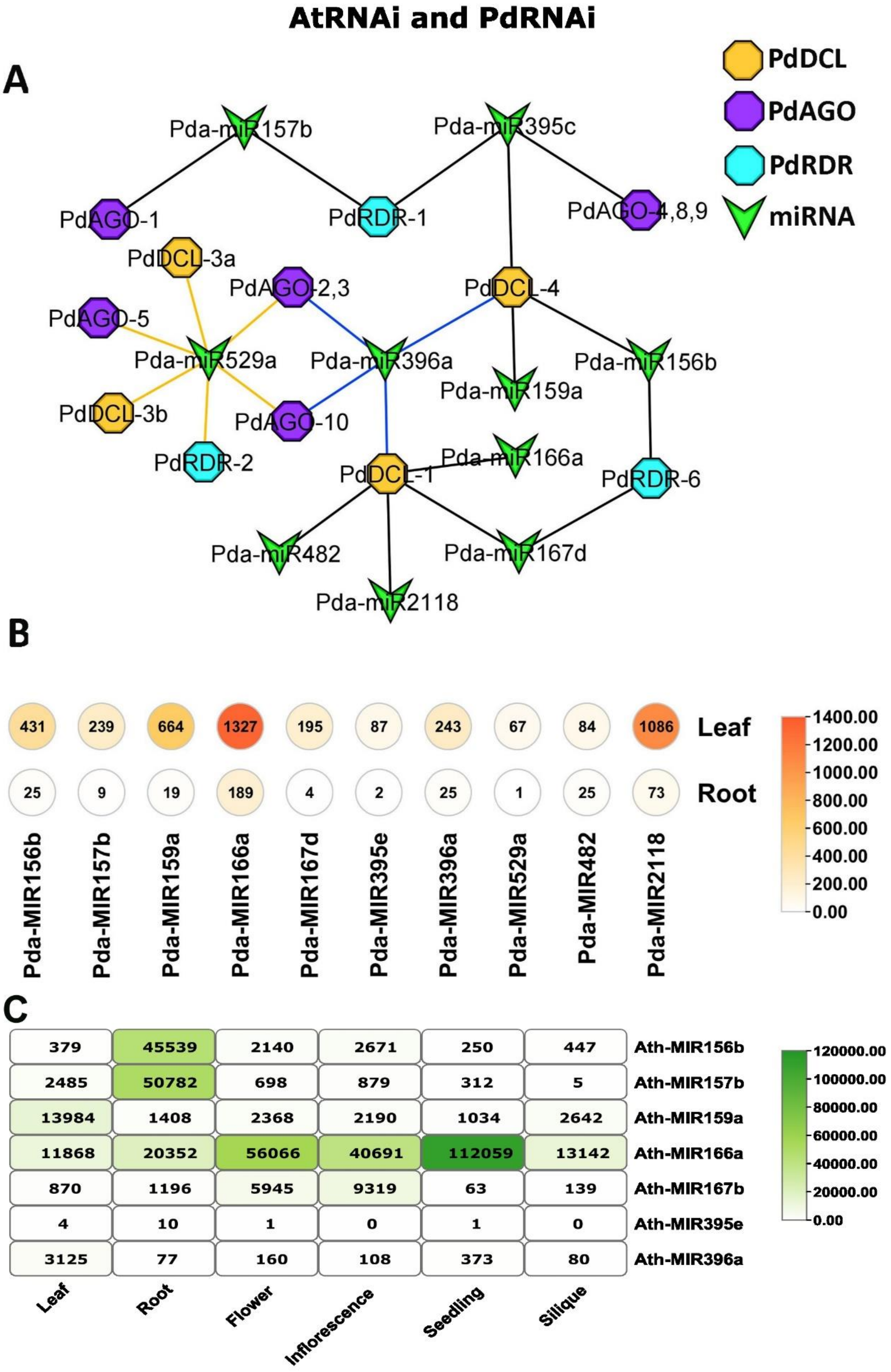
AtRNAi and PdRNAi
A
PdDCL
PdAGO
PdRDR
miRNA
Pda-miR157b
Pda-miR395c
PdAGO-1
PdRDR-1
PdAGO-4,8,9
PdDCL-3a
PdAGO-2,3
PdDCL-4
PdAGO-5
Pda-miR529a
Pda-miR396a
Pda-miR156b
Pda-miR159a
PdDCL-3b
PdAGO-10
Pda-miR166a
PdRDR-2
PdDCL-1
PdRDR-6
Pda-miR482
Pda-miR167d
Pda-miR2118
B
Leaf: 431 239 664 1327 195 87 243 67 84 1086
Root: 25 9 19 189 4 2 25 1 25 73
Pda-MIR156b
Pda-MIR157b
Pda-MIR159a
Pda-MIR166a
Pda-MIR167d
Pda-MIR395e
Pda-MIR396a
Pda-MIR529a
Pda-MIR482
Pda-MIR2118
1400.00
1200.00
1000.00
800.00
600.00
400.00
200.00
0.00
C
379 45539 2140 2671 250 447 Ath-MIR156b
2485 50782 698 879 312 5 Ath-MIR157b
13984 1408 2368 2190 1034 2642 Ath-MIR159a
11868 20352 56066 40691 112059 13142 Ath-MIR166a
870 1196 5945 9319 63 139 Ath-MIR167b
4 10 1 0 1 0 Ath-MIR395e
3125 77 160 108 373 80 Ath-MIR396a
Leaf
Root
Flower
Inflorescence
Seedling
Silique
120000.00
100000.00
80000.00
60000.00
40000.00
20000.00
0.00

Fig. S7A: miRNA associated with AtRNAi and PdRNAi. DCLs, AGOs, and RDRs are represented by light orange, purple, and pest colors, respectively and miRNAs are presented by light green color. B: The degree of miRNA expression in date palm leaf and root, are represented by deep to light orange colour. C: *A. thaliana* miRNA expression.

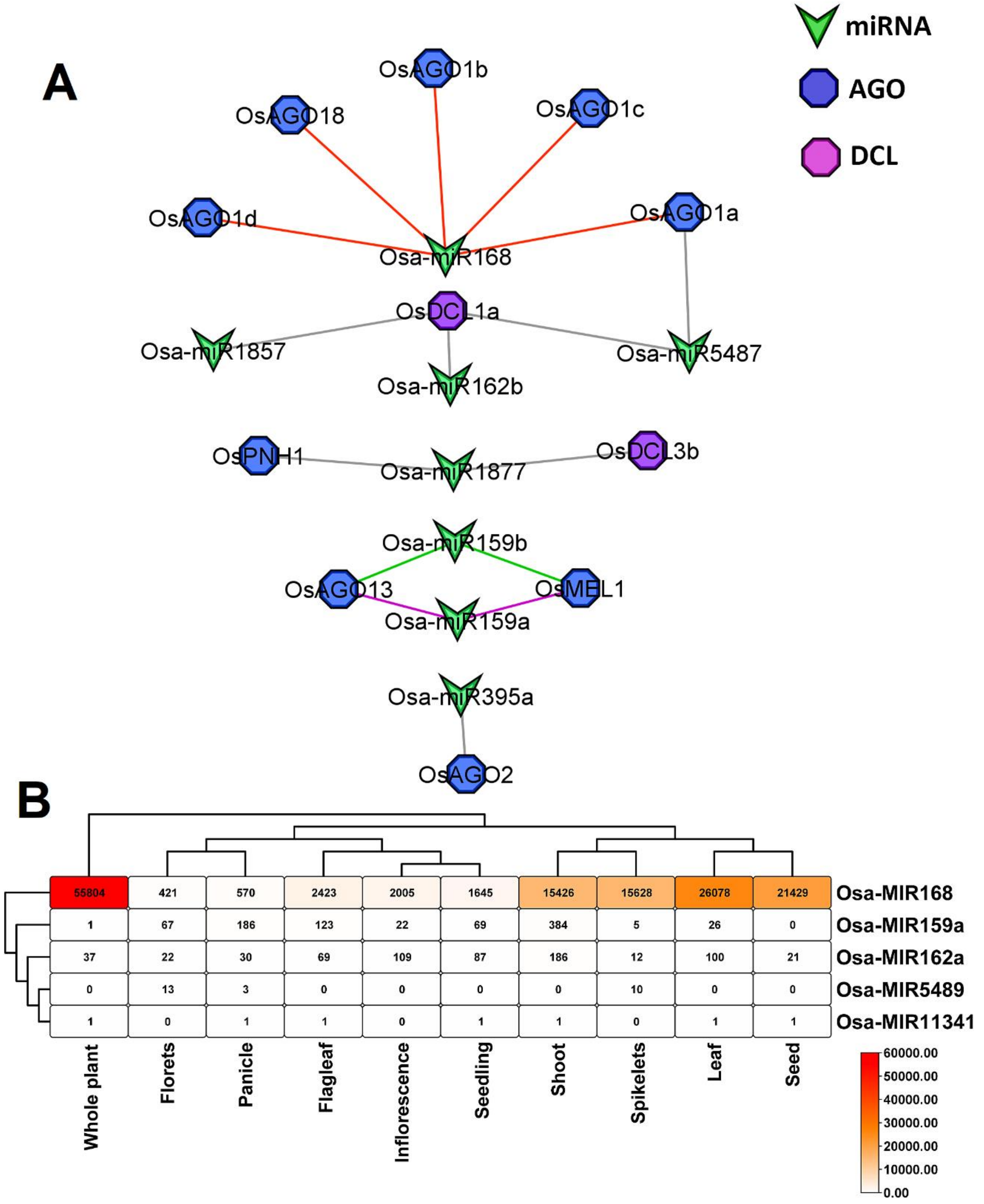

Fig. S7B: miRNA associated with OsRNAi. (A) The regulatory network among the miRNA and OsRNAi genes. (B) miRNA expression level in different plant organs of OsRNAi genes. DCL and AGO genes are represented by purple and blue color, respectively and miRNAs are presented light green color.
